# Uncertainty-aware electrification of university campuses: Matching demand with renewable generation for zero emissions^☆^

**DOI:** 10.1016/j.jestch.2026.102486

**Published:** 2026-09

**Authors:** César Berna-Escriche, Lucas Álvarez-Piñeiro, Paula Bastida-Molina, David Blanco-Muelas

**Affiliations:** a Instituto Universitario de Investigación en Ingeniería Energética (IUIIE), Universitat Politècnica de València (UPV), Camino de Vera s/n, 46022 Valencia, Spain; b Departamento de Estadística e Investigación Operativa Aplicadas y Calidad, Universitat Politècnica de València (UPV), Camino de Vera S/N, 46022 Valencia, Spain; c Departamento de Ingeniería Eléctrica, Universitat Politècnica de València (UPV), Camino de Vera 14 Camino de Vera S/N, 46022 Valencia, Spain

**Keywords:** Statistical analysis, BEPU Analysis, Wilks Methodology, Sustainable Energy Transition, University Campus decarbonization

## Abstract

Urban decarbonization requires robust methodologies to match electrified demand with on-site renewable generation under operational variability. University campuses are particularly well-suited testing environments, as their predominantly daytime demand profiles favor solar photovoltaic (PV) self-consumption. However, most campus-scale studies adopt deterministic approaches and do not explicitly quantify the impact of uncertainty in solar generation and demand, which can significantly affect system performance and design. This study proposes a structured and transferable methodology for campus decarbonization, integrating high-resolution hourly demand analysis, PV generation modeling, electrification measures, and uncertainty quantification within a Best Estimate Plus Uncertainty (BEPU) framework using Wilks’ non-parametric tolerance intervals. The approach is applied to the ETSII buildings at the Universitat Politècnica de València (UPV), within Valencia’s climate-neutrality strategy. For a case study with ∼3 GWh annual demand and 1.3 MWp PV capacity, results indicate that solar PV alone can supply about 70% of annual demand, with self-consumption levels of 67–69% and overall self-sufficiency near 50%. Adding a 1 MW / 4 MWh battery increases self-consumption above 95% and self-sufficiency to ∼65%, despite storage losses. The BEPU/Wilks framework provides statistically robust performance bounds, supporting reliable PV–storage sizing. The methodology provides a robust and scalable framework for planning the decarbonization of urban areas and university campuses.

## 1 Introduction

The global demand for energy has been steadily increasing over the past few decades. [1], having more than tripled in the last 50 years. Currently, fossil fuels account for approximately 80% of total primary energy production [2], creating a dual challenge. On the one hand, the ongoing depletion of these resources could, in the medium term, compromise the reliability of the electricity supply [3]. On the other hand, the continued reliance on fossil fuels drives the unsustainable rise in greenhouse gas (GHG) emissions [4], [5]. Consequently, pursuing decarbonization has become an essential priority across all sectors. Then, society must not only face the GHG emission problem but also the energy Trilemma, which remains a critical lens for understanding and steering global energy transitions. Effective management requires balancing rapid energy decarbonization with affordability and security considerations, while adapting strategies to local conditions, employing innovative technology and upholding principles of justice and sustainability [4].

Decarbonization strategies must prioritize sectors based on both their substantial contribution to GHG emissions and the complexity of required mitigation measures [6]. Major emitting sectors, including energy (25–30%), transport (15–20%), industry (15–20%), agriculture and land use (15–20%), and buildings (10–15%), require parallel and coordinated actions. While International Energy Agency roadmaps [7] emphasize early decarbonization of the power sector to enable widespread electrification, all sectors demand simultaneous attention due to their significant contributions. In particular, buildings and distributed end-use sectors deserve focused action, as their diffuse yet widespread emissions, linked to heating, cooling, domestic hot water, and appliances, offer cost-effective mitigation potential through electrification (e.g., heat pumps) and energy efficiency improvements.

Cities play a leading role in this transition, accounting for around 70% of global GHG emissions and concentrating demand across buildings, mobility, and energy systems [8]. This centrality makes them key platforms for integrated decarbonization strategies. Accordingly, transversal measures combining efficiency, electrification, and digitalization are essential to achieving climate neutrality targets, such as those established under the European Green Deal [9].

Key urban decarbonization strategies include the electrification of mobility through electric buses, expanded cycling infrastructure, and Electric Vehicle (EV) charging networks; improvements in building efficiency via retrofitting, heat pump deployment, and enhanced insulation; and the development of local energy communities enabling rooftop solar self-consumption and smart grid integration [10]. Additional measures involve advancing circular economy practices through waste valorization and green infrastructure, as well as leveraging digitalization, particularly Internet of Things (IoT) technologies, for real-time emissions monitoring and smart building management. Furthermore, the implementation of net-zero districts combines advanced solutions such as green hydrogen integration and high-efficiency lighting systems. Collectively, these coordinated actions address the majority of urban GHG emissions, supporting the transition towards climate-neutral cities by 2050.

### 1.1 State of the Art in Urban-Scale decarbonization

A literature review of recent publications on the urban scale collectively frames decarbonization as an inherently multisectoral challenge that requires the simultaneous transformation of buildings, energy infrastructure, mobility, and governance. At the building scale, Qiu et al. [11] and Zhu et al. [12] demonstrate that combining passive technologies (phase-change materials, radiative cooling, advanced insulation) with active distributed energy systems (PV, HP, combined cooling-heating-and-power) can reduce building energy demand by up to 50%, with Mixed-Integer Linear Programming (MILP)-based optimization models identified as the dominant tool for system design. Yuan et al. [13] further quantify the decarbonization potential of rooftop solar in commercial and industrial buildings, while Simpson et al. [14] demonstrate that transitioning district heating and cooling systems from fossil-fuel-based first-generation networks to fifth-generation ambient-temperature systems can significantly lower carbon emissions. These advanced systems leverage geothermal storage, waste heat recovery, and bidirectional heat flows. This approach reduces both on-site and grid-level GHG emissions. For instance, Stanford University's district energy retrofit achieved a 65% reduction in GHG emissions.

At the community and city scale, Belloni et al. [15] and Basaran et al. [16] highlight renewable energy communities (RECs) as a key organizational and technical model for scaling distributed renewables, with Net-Zero Energy Buildings acting as active grid-flexibility assets within these communities. The global diffusion of RECs remains uneven across regions. Northern Europe prioritizes large-scale wind and biomass installations. Meanwhile, Southern Europe, particularly Italy and Spain, experiences rapid growth in small-scale PV-based communities. This regional divergence is driven by national incentive frameworks. Abdelghany et al. [17] extend the decarbonization toolkit by incorporating green hydrogen as a key solution. They argue that while production costs remain the primary barrier (3.5–6.0 USD/kg in 2025), hydrogen enables decarbonization in hard-to-electrify urban sectors. These include long-duration grid storage, heavy transport, and district heating through hydrogen blending or dedicated networks. Digital infrastructure, such as IoT and digital twins, is identified as a critical enabler for smart urban hydrogen systems.

Governance and policy analyses highlight a persistent gap between ambitious climate targets and implementation capacity [10], [18], [19]. Key shortcomings include the lack of formalized climate budgets, incomplete sectoral decarbonization strategies, and limited consideration of scope 3 emissions. Current policy approaches are often dominated by subsidies and financial incentives, with insufficient cross-sector coordination [18]. Moreover, achieving climate neutrality requires embedding climate justice and energy equity as core elements of urban planning [19]. Overall, these findings emphasize the need for integrated governance frameworks that combine technical robustness, economic feasibility, and social inclusivity.

Urban decarbonization research is closely linked to university-scale studies, as campuses act as urban microcosms and serve as key testbeds for developing and scaling multisectoral decarbonization solutions [20]. Both domains require the coordinated transformation of buildings, energy systems, and governance frameworks, combining efficiency, renewable integration, and inclusive climate planning.

At the international level, universities have shown a growing commitment to sustainability through operational and educational measures. Amaral et al. [21] conducted an extensive *meta*-analysis of 424 university initiatives worldwide, classifying them into eight domains: Buildings, Energy, Air and Climate, Food, Transportation, Waste, Water, and Gardens. The results reveal that the most prevalent actions occur in the Energy and Buildings categories, which together account for more than half of the total initiatives analyzed. The Energy domain is characterized by the deployment of renewable energy systems, with solar PV and solar thermal technologies being the most prevalent. These actions not only reduce direct emissions but also contribute to the decentralization and diversification of campus energy systems. Furthermore, around 50% of electricity consumption in European university campuses corresponds to their total building demand.

Researchers have focused on this category, emphasizing the enhancement of energy efficiency in university campuses through retrofitting projects, advanced insulation materials, intelligent building control systems, and the phase-out of fossil-fuel-based heating systems. The transformative potential of these measures is amplified when combined with occupant awareness programs and behavioral interventions that encourage rational energy use, and universities should lead the way [22].

Recent literature has also examined the sociotechnical dynamics underlying sustainability transitions within universities [23]. Organizational factors, such as the commitment of institutional leadership, integration of sustainability in governance frameworks, and the existence of internal funding mechanisms, strongly influence the long-term success of decarbonization strategies. Similarly, the engagement of students and staff acts as a catalyst for cultural change, transforming energy-saving practices into durable habits. Universities, therefore, function not merely as consumers or producers of energy but as agents of societal transformation capable of bridging technological innovation with behavioral adaptation.

Some other studies have focused on the analysis of electricity consumption in university campuses, demonstrating that very few have developed this analysis, considering at the same time [24]: real data, 1-hour data sample period (or less), building analysis, and pattern analysis. Ortiz-Peña et al. did it [25] for the whole University of Castilla-La Mancha, while Aguayo-Ulloa [26] did it for the University of Bordeaux, although just considering 30 buildings on its Science and Technology campus. However, after their analysis, these studies do not include measurements to enhance energy sustainability transition.

Hurtado-Pérez et al. [27] focus on this issue and present a multicriteria methodology to evaluate the inclusion of solar PV installations in university campuses, taking UPV (Spanish acronym for Polytechnic University of Valencia) as a case study. Li et al. [28] investigated the performance and potential of various types of PV-assisted Heating, Ventilation, and Air Conditioning (HVAC) systems in a university in Wuhan. Pierce et al. [20] went a step further and proposed a digital twin model for analyzing decarbonization strategies of University College Dublin, modeling thermal and electricity networks, and including renewable generation based not only on solar PV, but also on biomass support.

In all of them, solar PV inclusion arises as necessary, supporting findings of [23] about the importance of the sustainability transition in university campuses. Nevertheless, the operation of solar-based systems is intrinsically subject to uncertainty, which remains unexplored in most of the studies despite its importance. Solar irradiance fluctuates by both diurnal and seasonal factors, while PV performance may degrade over time due to temperature effects, module soiling, and material aging [29], [30].

Moreover, load profiles in high academic buildings exhibit temporal variability associated with class schedules, research cycles, and occupancy levels. Addressing these uncertainties requires advanced modeling techniques that incorporate stochastic input variables to assess expected system performance under different scenarios [5], [31]. The literature review demonstrates that this issue remains unexplored, despite its importance when considering renewable generation, as the generation-consumption match is completely necessary. Thus, probabilistic simulation, sensitivity analysis, and uncertainty quantification play key roles in this process, providing a scientific basis for robust design and decision-making.

To better position this study with respect to the existing literature, Table 1 summarizes the most closely related contributions on campus energy modeling, PV integration, and uncertainty treatment. Particular attention is given to whether the studies rely on deterministic or stochastic approaches and whether uncertainty is explicitly propagated in the assessment of PV-demand matching.

**Table 1:** Summary of closely related studies on campus energy modeling and PV integration, with emphasis on deterministic versus stochastic approaches and explicit uncertainty treatment.

Reference	Case study	Focus	Modeling approach
Ortiz-Peña et al. [25]	University of Castilla-La Mancha	Analysis of electricity consumption patterns across a university system	Deterministic
Aguayo-Ulloa [26]	University of Bordeaux (30 buildings, Science and Technology campus)	Building-level/campus electricity consumption analysis	Deterministic
Hurtado-Pérez et al. [27]	Polytechnic University of Valencia	Multicriteria methodology for PV integration in a university campus	Deterministic
Li et al. [28]	University campus in Wuhan	Performance and potential of PV-assisted HVAC systems	Deterministic
Pierce et al. [20]	University College Dublin	Digital twin for campus decarbonization, including thermal and electrical networks and renewable generation	Deterministic
Previous BEPU/Wilks applications [5], [31]	Energy systems / non-campus applications	Uncertainty quantification using BEPU/Wilks methods	Stochastic
This work	ETSII buildings, Polytechnic University of Valencia	Campus-scale PV-demand matching, electrification, storage sizing, and uncertainty-aware decarbonization assessment	Deterministic + stochastic (BEPU/Wilks)

### 1.2 Research objectives and scope of the study

As shown in Table 1, conventional analyses of energy systems in general, and particularly those conducted at the urban scale, typically rely on deterministic approaches based on single-objective optimization [32], [33], [34]. Such methods often fail to adequately capture the inherent variability associated with renewable energy generation and demand profiles. The methodology proposed in this research specifically addresses these limitations in urban-scale decarbonization analyses. The proposed approach overcomes these gaps through a novel integrated framework that combines:

(1) Full end-use electrification, including the evaluation of potential measures such as the elimination of fossil-fuel-based energy uses, the deployment of HP, and the utilization of waste heat for DHW production;

(2) The optimized design of renewable energy systems, including the estimation of the available generation potential and the optimal sizing of energy storage systems; and.

(3) A comprehensive performance assessment that considers deterministic values together with the impact of uncertainties affecting both generation and demand profiles. This uncertainty analysis is performed using BEPU methodologies and Wilks’ order statistics, which bound the system's variability at predefined coverage and confidence levels.

The major novelty relies on the combined use of BEPU-Wilks framework at the building level, because it brings a safety–grade uncertainty methodology into the micro–scale domain of building decarbonization, where analyses are still predominantly deterministic. Instead of sizing and operating building energy systems (PV, storage, heating, cooling, etc.) from a single “typical” scenario, the method delivers statistically justified performance bounds (e.g., on self-consumption, emissions, or unmet load) under realistic variability of weather, occupancy, and equipment behavior, which typical deterministic building life cycle assessments (LCAs) and energy models ignore.

Methodologically, a best–estimate building model is first created, and then key uncertain inputs (climate, loads, technology performance) are sampled; Wilks’ non–parametric formula is used to determine how many runs are needed to obtain tolerance intervals with given coverage and confidence, without assuming any specific probability distribution and with far fewer simulations than full Monte Carlo. Applied to building decarbonization, this provides decision–makers with guaranteed “worst–case” and “best–case” operational and emissions outcomes, reveals when deterministic designs are overly optimistic or fragile, and supports more robust choices of on–site renewables and storage that remain effective across many plausible futures rather than a single design day or reference year. The proposed analysis relies on hourly modeling of both solar PV generation and building energy consumption. This modeling framework is driven by validated meteorological datasets and detailed operational load profiles, enabling the development of adaptive strategies that ensure reliable system performance under variable operating conditions. The methodology follows an evidence-based process that integrates data collection, diagnostic energy audits, scenario modeling, and stochastic evaluation in order to identify robust and context-specific decarbonization pathways.

This methodology was applied to the UPV, specifically to the campus located in the city of Valencia (Spain), known as the Vera Campus. The objective of this work aligns with the broader goals of the European initiative “Missions Valencia 2030”. Valencia has been identified by the European Commission as one of the 100 European cities committed to achieving climate neutrality by 2030 [35]. In response, the city has adopted this vision through the “Missions Valencia 2030” initiative, embedded within the “Valencia Urban Strategy 2030” [36].

Building on this foundation, the main objectives of this research are defined as follows:

1. Quantify robust decarbonization pathways under uncertainty, ensuring reliable performance (e.g., 95% coverage, 95% confidence via Wilks' order statistics) through the propagation of variability in PV generation and demand to the system energy balances.
2. Optimize integrated system design, combining full electrification (HP, fossil fuel phase-out), PV sizing, and storage capacity to maximize self-consumption and minimize grid dependence.
3. Deliver context-specific strategies through hourly modeling with validated meteorology and real operational profiles, enabling adaptive and scalable solutions.
4. Align with policy targets by providing evidence-based pathways replicable across Valencia's demonstration districts to achieve climate neutrality by 2030.

Therefore, the main findings of this study can be viewed from three perspectives. From a technical standpoint, it is a pioneer in the building-scale application of the BEPU-Wilks methodology, which extends uncertainty quantification to the field of urban decarbonization and requires only a reduced number of simulations to obtain robust performance limits (usually at 95%/95% coverage and confidence levels), compared to the thousands required by the Monte Carlo method. From a policy perspective, it offers statistically guaranteed pathways that align with the climate-neutrality objectives and European and global decarbonization goals. From a practical standpoint, building operators gain interactive digital twins that display risk-adjusted performance (e.g., variability in the performance of the combination of PV energy and energy storage systems, as well as that associated with load uncertainties), replacing the fragile deterministic predictions of EnergyPlus with resilient investment decisions.

To develop all commented aspects and achieve the stated objectives, the document is organized as follows: Section 2 details the methodological approach; Section 3 presents the case study of the ETSII (Spanish acronym for Higher Technical School of Industrial Engineers) buildings located on the Vera campus of UPV; Section 4 discusses the results and implications of the analysis; and Section 5 concludes by outlining the broader potential for replication and policy integration within European urban campuses.

## 2 Methodology

### 2.1 Methodological framework

The proposed methodological framework provides a structured approach to support the decarbonization of energy systems through evidence-based analysis and strategic planning. While deterministic energy balance simulations dominate building decarbonization studies, balancing fixed renewable generation (PV output from TMY data) against loads using tools like EnergyPlus or TRNSYS [37]. These approaches produce single-point predictions and are therefore vulnerable to performance differences due to unmodeled climate variability and demand. The BEPU-Wilks methodology addresses this gap by transferring nuclear safety-grade uncertainty quantification to building/urban renewable systems [38], [39], providing statistically robust tolerance bounds (e.g., 95%/95% coverage via 59 simulations) without parametric distribution assumptions. Precedents exist in island-scale applications (Canary Islands 2040 forecasts [5], [31]) but remain novel at the building level, where deterministic baselines persist.

The methodology integrates data collection, diagnostic assessment, scenario modeling, and uncertainty evaluation to support informed decision-making. A key contribution of this study lies in the explicit treatment of uncertainty, addressing variability in renewable generation and demand through stochastic methods such as Wilks’ tolerance intervals. The comprehensive modeling of energy systems for decarbonization is structured around three fundamental phases, combining deterministic diagnostics, scenario simulation, and robust validation. The main phases of the analysis are therefore defined as follows:

1. Data collection and deterministic baseline

The methodology begins with a review of successful low-carbon implementations in analogous facilities, combined with the deployment of high-resolution monitoring (hourly or sub-hourly electricity and thermal demand profiles) to characterize load variability, peak demand, and system inefficiencies [40], [41]. A deterministic energy balance model is then formulated using validated simulation platforms (e.g., EnergyPlus, TRNSYS, HOMER Pro, or EnergyPLAN), where PV generation is represented under fixed boundary conditions [42], [43].

2. Measure identification and scenario simulation

A set of technically feasible interventions (such as heat pumps, building envelope improvements, solar PV systems, battery storage, and demand-response strategies) should be identified and evaluated using multi-criteria analysis based on techno-economic and environmental indicators (e.g., LCOE, NPV, CO_2_ abatement costs) [44]. Alternative system configurations (e.g., PV-only, PV + BESS, PV + HP + BESS) can then be assessed through high-resolution (8760-hour) simulations to optimize dispatch and maximize self-consumption under realistic demand profiles, renewable resource variability, and tariff structures [45].

3. BEPU methodology for uncertainty quantification

The BEPU methodology combines a deterministic best-estimate energy model with a systematic and quantitative treatment of uncertainty [46]. It begins with a detailed energy balance model representing the most probable system behavior, and then propagates key uncertainties related to weather conditions, demand profiles, and system performance [5]. This helps achieve a more realistic representation of variability in renewable energy systems. Uncertainty propagation is performed using Wilks’ non-parametric tolerance interval method [47], which allows the estimation of statistically robust performance bounds without assuming predefined probability distributions. For instance, approximately 93 simulations are sufficient to obtain two-sided tolerance intervals with 95% coverage and 95% confidence, significantly reducing computational requirements compared to full Monte Carlo approaches [48]. Instead of producing a single deterministic outcome, the methodology generates performance ranges, enabling the evaluation of key indicators such as self-consumption, unmet load, and grid dependency under uncertain operating conditions. This approach provides a more robust basis for system design and supports risk-informed decision-making.

While each stage is essential to ensure a comprehensive and coherent analysis, particular emphasis is placed on phase 3, corresponding to the BEPU methodology. Phase 1 establishes the initial baseline scenario through the use of validated simulation tools. Phase 2 then evaluates potential improvement measures and alternative system configurations. However, phase 3 constitutes the core of the approach. It not only allows the estimation of the system’s best-estimate performance, but also provides an accurate quantification of the uncertainties affecting the energy balances. This allows for the derivation of statistically robust performance bounds, ensuring a reliable assessment of system behavior under uncertainty. Therefore, phase 3 will be described in greater detail in the following subsections.

### 2.2 BEPU methodology for uncertainty quantification

As previously outlined, the quantification of uncertainty and the assessment of system robustness are central objectives of this study. To address this, the BEPU methodology is applied. The approach combines deterministic modeling (the most likely system behavior), with structured quantification of uncertainties, enabling a comprehensive evaluation of system resilience and performance under varying conditions.

Originally developed for thermal–hydraulic safety assessments [39], BEPU has been extensively validated and is now widely applied across industrial and research domains. While deterministic methods provide single-point estimates based on fixed assumptions, BEPU extends this framework by generating statistically bounded outputs, thus supporting more reliable risk and performance evaluations. Conceptually, BEPU integrates deterministic and probabilistic analyses, commonly referred to as deterministic safety assessment (DSA) and probabilistic safety assessment (PSA), respectively [49].

In contrast to deterministic approaches, stochastic methods explicitly account for variability in real-world systems, including both aleatoric (intrinsic randomness) and epistemic (knowledge uncertainty) components [31]. This is particularly relevant in renewable-based energy systems, where generation and demand profiles exhibit significant temporal variability. Deterministic analyses often neglect these effects, leading to either over-conservative designs or underestimation of system requirements [5].

To address these limitations, BEPU is coupled with Wilks’ non-parametric order statistics, which provide a computationally efficient and distribution-free method for uncertainty quantification. Wilks’ formula determines the minimum number of simulations required to construct tolerance intervals with predefined confidence and coverage levels, significantly reducing computational effort compared to conventional Monte Carlo methods [50]. This approach is especially suitable for energy balance analysis, where scalar performance indicators (e.g., annual generation-demand balance) can be derived from each simulation, ensuring compliance with the statistical assumptions required for its application [38].

Overall, the BEPU–Wilks framework allows the derivation of statistically robust performance bounds, providing a reliable basis for the design and operation of energy systems under uncertainty, and extending established best practices from safety-critical industries to the field of urban energy system modeling.

#### 2.2.1 Wilks’ statistical method

In 1941, Wilks proposed a statistical methodology to determine the minimum sample size required to establish two-sided tolerance limits with a specified confidence level [50]. This method, widely known as Wilks’ formula, has gained increasing relevance in modern computational applications, particularly within the nuclear industry. It has been formally adopted by the U.S. Nuclear Regulatory Commission (NRC) as a basis for licensing decisions involving BEPU analyses [51]. As a result, several major organizations in the nuclear sector, including GRS [52] and Westinghouse [53], have implemented this approach in their analytical frameworks. More recently, the publication of good practice guidelines for uncertainty quantification in nuclear engineering has further emphasized the method’s importance and consolidated its role as a standard tool in safety assessment and regulatory processes [54].

Subsequently, Wilks extended his analytical framework to include one-sided tolerance limits [47], while Wald further generalized its applicability to multivariate distributions [55]. The Wilks method is designed to quantify uncertainty through the construction of tolerance intervals, defined as regions expected to contain a specified proportion (γ) of the true values of a variable with a given confidence level (β). Here, γ represents the coverage (the fraction of possible true outcomes included by the interval) and β denotes the confidence level (probability that such coverage is achieved).

Two types of tolerance intervals are distinguished: one-sided intervals, expressed as (-∞,U] or [L,∞), corresponding to output variables constrained to remain below or above certain thresholds; and two-sided intervals, expressed as $\left[L,U\right]$, representing variables that must remain within predefined bounds. The Wilks method estimates these intervals by performing n simulations in which uncertain input parameters are stochastically varied, with the minimum and maximum observed values defining the interval limits. The required number of simulations directly depends on the desired coverage and confidence levels, as larger values demand a greater computational effort to achieve statistically robust results.

Accordingly, the number of simulations (n) necessary for constructing one-sided tolerance intervals is defined by [47]:

(1) $$n=\min\left\{n\in N|1-\gamma^{n}\geq \beta \right\}$$

Being γ the coverage (the fraction of possible true outcomes included by the interval), and β the confidence level (probability that such coverage is achieved).

In contrast, the determination of two-sided tolerance intervals is performed using the following expression [50]:

(2) $$n=\min\left\{n\in N|\left(n-1\right)\gamma^{n}-n\gamma^{n-1}+1\geq \beta \right\}$$

For the typical coverage and confidence levels employed in many industrial applications, namely 95% for coverage and confidence levels, the required number of simulations to construct tolerance intervals is obtained after clearing up n in Eqn. (1) or Eqn. (2) for one-sided or two-sided cases, respectively, leading to 59 and 93 simulations. When both the coverage and confidence levels are increased to 99%, the computational demand rises substantially, requiring 459 simulations for one-sided intervals and 662 for two-sided intervals.

### 2.3 Computational implementation

Hourly electricity demand profiles are obtained from local measurement systems, stored in standardized formats (Excel, CSV), covering complete annual cycles of 8,760 h for non-leap years. Solar PV generation data are retrieved from the European Commission's PV Geographical Information System (PVGIS) v5.2 API [56], utilizing the SARAH2 satellite-derived database (covering the period from 2005 to 2020). PVGIS provides validated hourly irradiance components, power output, and meteorological data for user-specified geographic coordinates, system configurations (tilt angle, azimuth, losses), and PV technology types. The computational model is implemented in Python.

Following Wilks' method for two-sided tolerance intervals with 95% coverage and 95% confidence (Eqn. (2)), 93 independent solar irradiance profiles were generated by stochastically varying key PVGIS input parameters, including meteorological year selection, system loss coefficients, and temperature parameters. This sample size ensures that the resulting tolerance bounds capture 95% of potential system outcomes with 95% statistical confidence. Similarly, 93 demand profile variations were generated to account for consumption variability and operational uncertainty.

For each of the 93 sampled combinations, an hourly energy balance model implements a rule-based maximum self-consumption strategy over the complete annual horizon (on an hourly timestep t). At each hour, the dispatch algorithm prioritizes: (i) direct PV power supply to electrical load through DC-AC inversion (accounting for inverter efficiency η_inv_), (ii) battery charging from excess PV generation (subject to maximum capacity $C_{\mathrm{bat}}$ and power $P_{\max}$ constraints), and (iii) sequential supply from battery discharge or grid import to meet residual demand.

Battery state of charge dynamics is governed by Eqn. (3):

(3) $$SoC\left(t\right)=SoC\left(t-1\right)+\left(P_{\mathrm{charge}}\left(t\right)\cdot \eta_{\mathrm{bat}}-P_{\mathrm{charge}}\left(t\right)\right)\cdot \Delta t$$

Where SoC represents the state-of-charge of the battery, t is the time, P_charge_ indicates the instantaneous power of recharge, $\eta_{\mathrm{bat}}$ represents round-trip battery efficiency and Δt = 1 h. The constraint 0 ≤ $SoC\left(t\right)$ ≤ Cᵦ_at_ is enforced at all timesteps.

For each simulation run, key performance indicators are computed, including the self-consumption rate, self-sufficiency rate, and grid import/export flows. The 93 simulation results generate empirical distributions for each output variable, from which two-sided 95/95 tolerance intervals are constructed by identifying the minimum and maximum values across all runs.

### 2.4 BEPU Workflow implementation

**Fig. 1:**
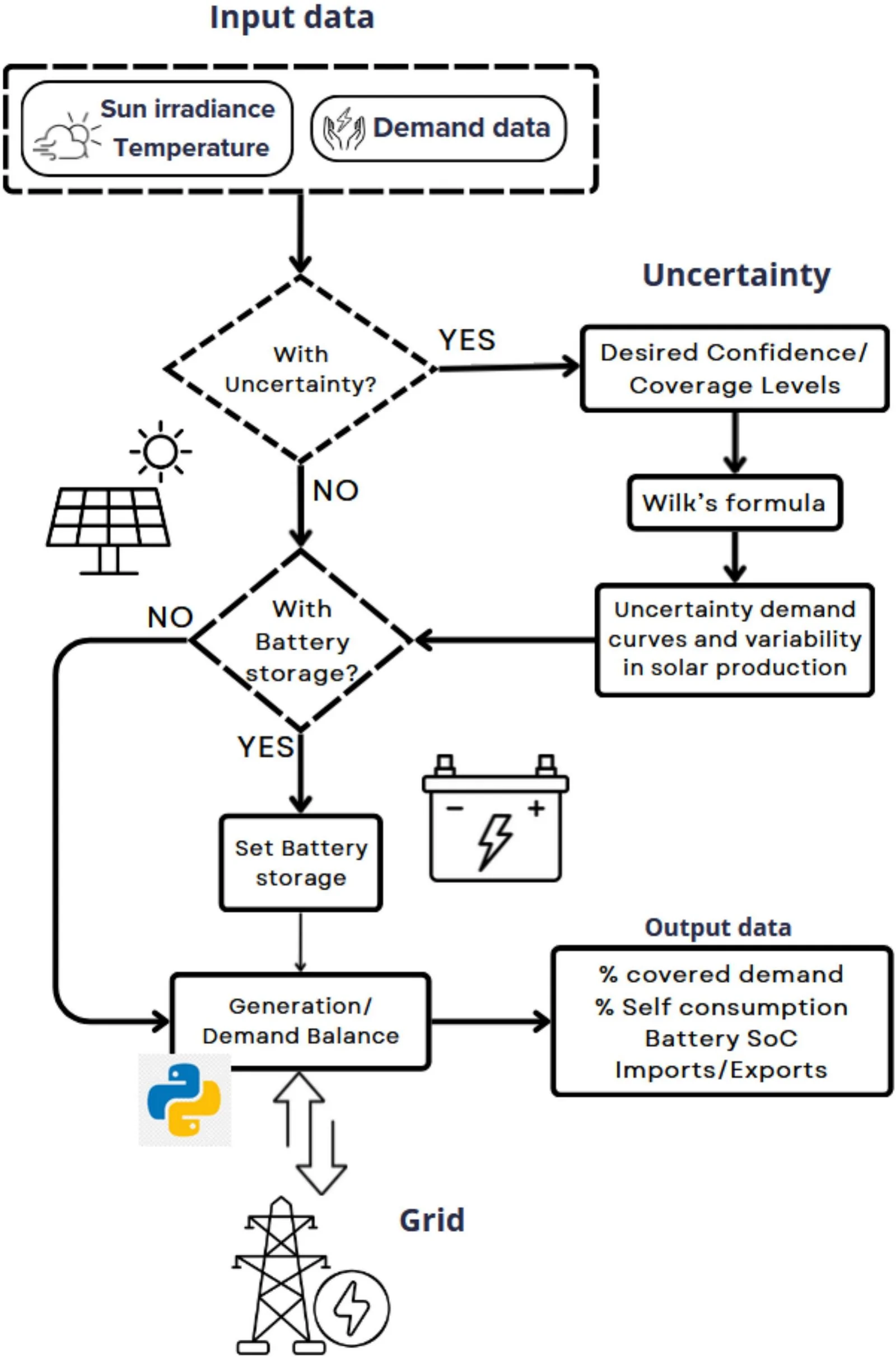
Methodology flowchart.

Specifically, after completing all the preliminary phases of problem contextualization, data collection, and analysis, the implementation process of the proposed methodological framework for the BEPU methodology is represented in more detail in Fig. 1.

In the current case, a BEPU methodology has been used, combining deterministic and probabilistic analyses to provide realistic predictions of system behavior along with quantified uncertainties. When the probability density functions (PDFs) of input variables are unknown, assumptions or expert-based estimates are often needed, introducing potential errors [48]. Non-parametric approaches, such as Monte Carlo sampling, avoid this by running numerous randomized simulations to derive output distributions, though at high computational cost. Wilks’ method offers a simplified alternative with reduced simulation requirements [[47], [50]].

## 3 Case study: ETSII buildings of the Vera campus from the UPV

To prove the feasibility of the methodology, it has been applied to the ETSII buildings of the Vera Campus UPV, in Valencia. Therefore, this section describes the regulatory framework associated with the study, along with the main characteristics of the aforementioned UPV buildings, which served as the initial reference for the development of the proposed methodology.

### 3.1 Valencia city and its Polytechnic university of Valencia

Valencia has been selected by the European Commission as one of 100 cities aiming to achieve climate neutrality by 2030 under the European Green Deal framework [35]. To address this objective, the city adopts a mission-oriented approach that prioritizes manageable urban subunits, such as districts, to enable targeted and scalable decarbonization strategies. This vision is implemented through the “Missions Valencia 2030” initiative, aligned with the Valencia Urban Strategy 2030.

In this context, the UPV acts as a living laboratory, supporting the development and validation of replicable decarbonization solutions. In particular, the Vera Campus, and specifically the ETSII buildings, serves as a pilot case for evaluating technical and economic feasibility. The strategy focuses on solar PV deployment, energy efficiency, and demand-side management. With an annual electricity demand of approximately 3 GWh, largely concentrated during daytime, the ETSII buildings exhibit strong compatibility with PV-based solutions.

### 3.2 Higher technical school of industrial engineering

ETSII is located at the Vera Campus of UPV. It comprises 18 buildings, with a total footprint area of 5,216 m^2^ and approximately 50,000 m^2^ of built area. Their buildings host multiple university centers and departments covering a wide range of engineering disciplines at both undergraduate and postgraduate levels. These include programs in industrial, chemical, energy, biomedical, and organizational engineering, among others. The faculty comprises approximately 746 academic and administrative staff and serves around 4,200 students annually [57].

A diagnostic framework is employed to define the measures analyzed in this study, using energy consumption data from the ETSII at UPV. The assessment covers all ETSII buildings, as well as associated facilities including a warehouse, greenhouses, a data centre, and a boiler plant for heating and domestic hot water. Metered data and building energy modeling are used to characterize baseline energy demand, while primary energy intensity (kWh/m^2^·year) highlights the dominant load components. In particular, thermal systems account for approximately 40–50% of total consumption, with data centre cooling emerging as another critical energy driver.

The diagnostic assessment identifies key strategic measures to optimize campus energy performance, building upon prior efficiency improvements such as LED retrofits. The focus shifts to advanced interventions, notably the replacement of natural gas boilers with reversible heat pumps (HPs) that assist in waste-heat recovery. This cascade configuration effectively matches opposing thermal demands, such as data centre cooling and seasonal building heating, achieving primary energy savings of 50–70% and aligning with successful large-scale implementations such as Stanford’s SESI project [[58], [59]].

In parallel, rooftop solar PV systems are deployed to maximize on-site renewable generation and self-consumption, leveraging the strong alignment between daytime demand and solar production. The integrated deployment of HPs and PV establishes a synergistic energy management framework, where cooling-driven waste heat is reutilized and PV electricity supports system operation, reducing grid dependency and enhancing system resilience [60].

Implemented as a pilot at ETSII, this modular approach combines real demand profiles and system simulations to validate technical and economic feasibility. The resulting framework provides a scalable and replicable pathway for campus decarbonization, with potential applicability to broader urban contexts. Subsequent analysis focuses on evaluating system performance under uncertainty, incorporating variability in both renewable generation and demand.

## 4 Results and discussion

This section presents the results obtained by applying the proposed methodology to the ETSII buildings at UPV, organized into three main subsections and a discussion section. First, a detailed analysis of the energy consumption patterns and the main characteristics of the initial system is carried out. The analysis then advances to Phase 2 (measure identification and scenario simulation) and Phase 3 (uncertainty quantification), which constitutes the core methodological contribution. In Phase 2, decarbonization measures, including high-efficiency heat pumps, building envelope retrofits, and solar PV systems with battery storage, are identified and prioritised using multi-criteria decision frameworks. Phase 3 evaluates alternative system configurations (e.g., PV-only, PV + BESS, PV + HP + BESS) through 8760-hour simulations, followed by the application of Wilks’ tolerance intervals to derive statistically robust performance bounds under uncertainties in weather and demand.

Phase 3 is further divided into deterministic and stochastic subphases. The deterministic analysis evaluates system performance under fixed conditions using a reference year (2019), whereas the stochastic analysis incorporates variability through long-term solar irradiance data (PVGIS) and demand fluctuations [56]. This allows the assessment of performance variability under realistic operating conditions.

### 4.1 Contextualization and analysis of the current energy consumption

This section initially delineates the current status of the ETSII buildings at the UPV, establishing a baseline assessment of energy consumption profiles, renewable generation capacities, and the associated emissions footprint (methodology − phase 1).

The analysis of all ETSII-associated buildings, together with the available data recorded by their installed meters, allows the buildings to be classified into four main categories: main buildings, greenhouses, the data center, and auxiliary buildings [58].

**Fig. 2:**
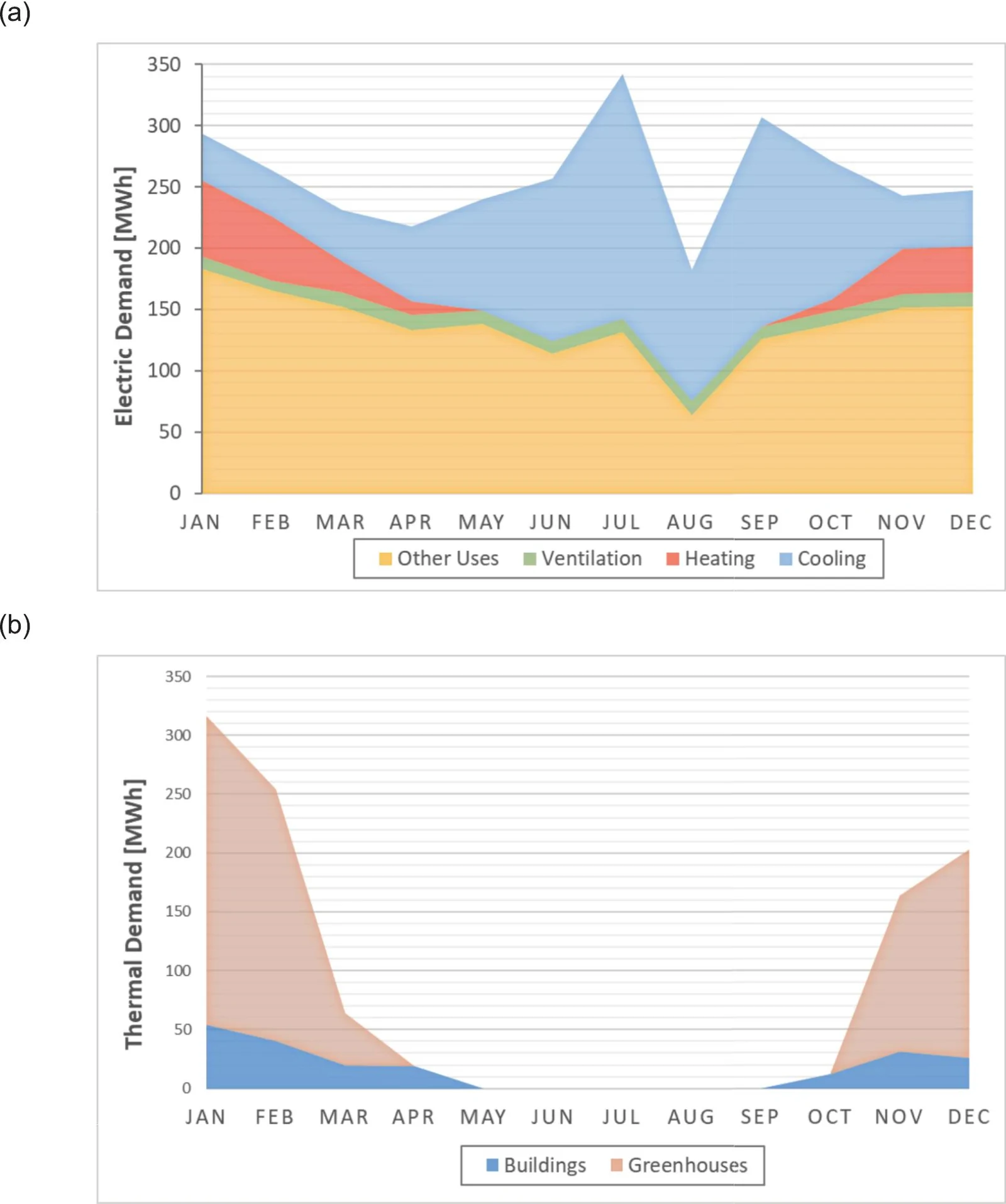
Monthly energy demands: (a) Electric demand; (b) Thermal demand (covered with gas boilers).

As shown in Fig. 2(a), the electrical demand has been disaggregated into four end uses: three associated with air conditioning (heating, cooling, and ventilation) and a fourth accounting for all the remaining uses, primarily lighting, consumption by computer and office equipment, and the main experimental and laboratory equipment employed for research, teaching activities, and maintenance operations. Regarding fossil fuels, there is still some consumption for space heating in several of the main buildings and in certain school-affiliated greenhouses; Fig. 2(b) presents the average monthly values corresponding to these contributions.

**Fig. 3:**
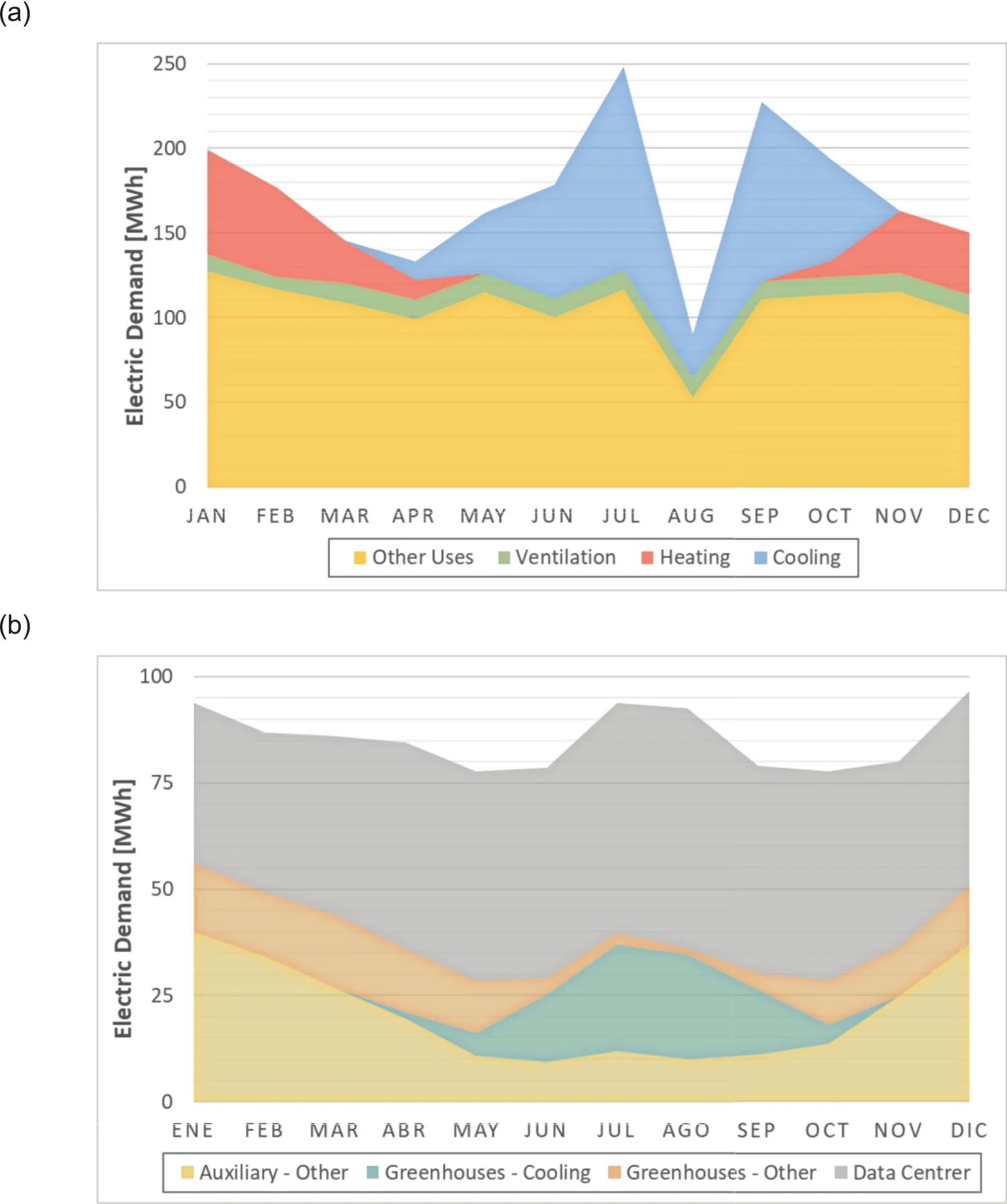
Electric Demand: (a) In main buildings, split into the four major categories. (b) In Auxiliary Buildings, Greenhouses, and Data Center.

To analyze building energy demands in greater detail, the electrical consumption profiles shown (Fig. 2-(a)) disaggregate into four categories by function. Main buildings account for the largest share of total energy demand, including classrooms, laboratories, offices, and administrative areas (Fig. 3-(a)). Energy consumption in these buildings includes air conditioning (heating, cooling, ventilation), as well as electrical loads (lighting, office equipment, computers, and specialized machinery). Operations exhibit stable daily patterns with consistent occupancy from 7:00 a.m. to 9:00p.m., resulting in regular load profiles characterized by a sharp morning peak due to sequential system start-ups.

The auxiliary buildings include a maintenance building, boiler room, and outdoor sports facilities, where energy consumption primarily supports lighting and small maintenance machinery (Fig. 3-(b) Auxiliary-Other). Operating hours align closely with those of the main buildings, typically from 7:00 a.m. to 9:00p.m. The greenhouses, affiliated with the school, exhibit distinctly higher and more variable energy demands driven by climate control and ancillary uses (Fig. 3-(b) Greenhouses). Cooling predominates in summer, while heating is essential in winter, often required continuously, especially overnight. The secondary load covers lighting, drip irrigation pumps, and minor tools. Air conditioning needs are highly seasonal and weather-dependent; currently met by gas boilers, this demand will be fully offset by waste heat from the data center, as detailed later.

The data center operates continuously year-round, maintaining steady cooling loads with minor seasonal variations (Fig. 3-(c) Data Center), peaking in summer, and diurnal fluctuations tied to ambient light and heat gains. Electrical consumption remains constant overall, reflecting uninterrupted information technology system requirements. Analyzing Fig. 2 and Fig. 3 it can be observed that currently, electricity dominates building energy use, totaling approximately 3.2 GWh annually: ∼2 GWh for main buildings, 0.25 GWh for auxiliary buildings, 0.5 GWh for the data center, and 0.2 GWh for greenhouses. Residual natural gas consumption persists, albeit limited to the heating of three of the main buildings and the greenhouses. This totals approximately 1 GWh (thermal energy), disaggregated as 0.2 GWh for the main buildings and 0.8 GWh for the greenhouses.

### 4.2 Decarbonizing measures and the stochastic nature of generation and demand

Following the initial analyses of the current system, section 4.2 addresses the major aspects of identifying and evaluating decarbonization strategies and studying the nature of the variables associated with energy generation and consumption. Phase 2 identifies and prioritizes measures by assessing interventions for technical feasibility, economic viability, and environmental impact, including high-efficiency equipment replacement, heating/cooling system optimization, building envelope upgrades, and renewable/hybrid energy deployment, supported by decision tools and multi-criteria analysis. This phase simulates alternative configurations by modeling energy supply and demand scenarios incorporating HP, solar PV, energy storage, and demand-side management, accounting for the stochastic nature of solar generation and demand profiles to determine optimal technology combinations aligned with facility operations and local resources.

#### 4.2.1 Identification and Prioritization of decarbonization measures

To improve energy efficiency, two principal measures were implemented. First, gas-fired heating systems were replaced with high-efficiency HP, eliminating natural gas consumption and the associated direct CO_2_ emissions from these sources. This intervention yields the predominant reduction in GHG emissions. Second, waste heat recovered from the continuously operating data center is utilized for building heating, proving especially effective from October to April when nearly all available thermal energy is exploited; minor surpluses persist only in transitional months such as April and October.

Prior gas boiler emissions were quantified using a natural gas CO_2_ emission factor of 0.252 kg CO_2_ per kWh of final energy, totaling approximately 260 tons CO_2_ annually. Although heat pump substitution delivers the largest direct emissions cut, data center waste heat integration substantially offsets heating demand and curtails electricity consumption at negligible marginal cost, enhancing overall system efficiency.

**Fig. 4:**
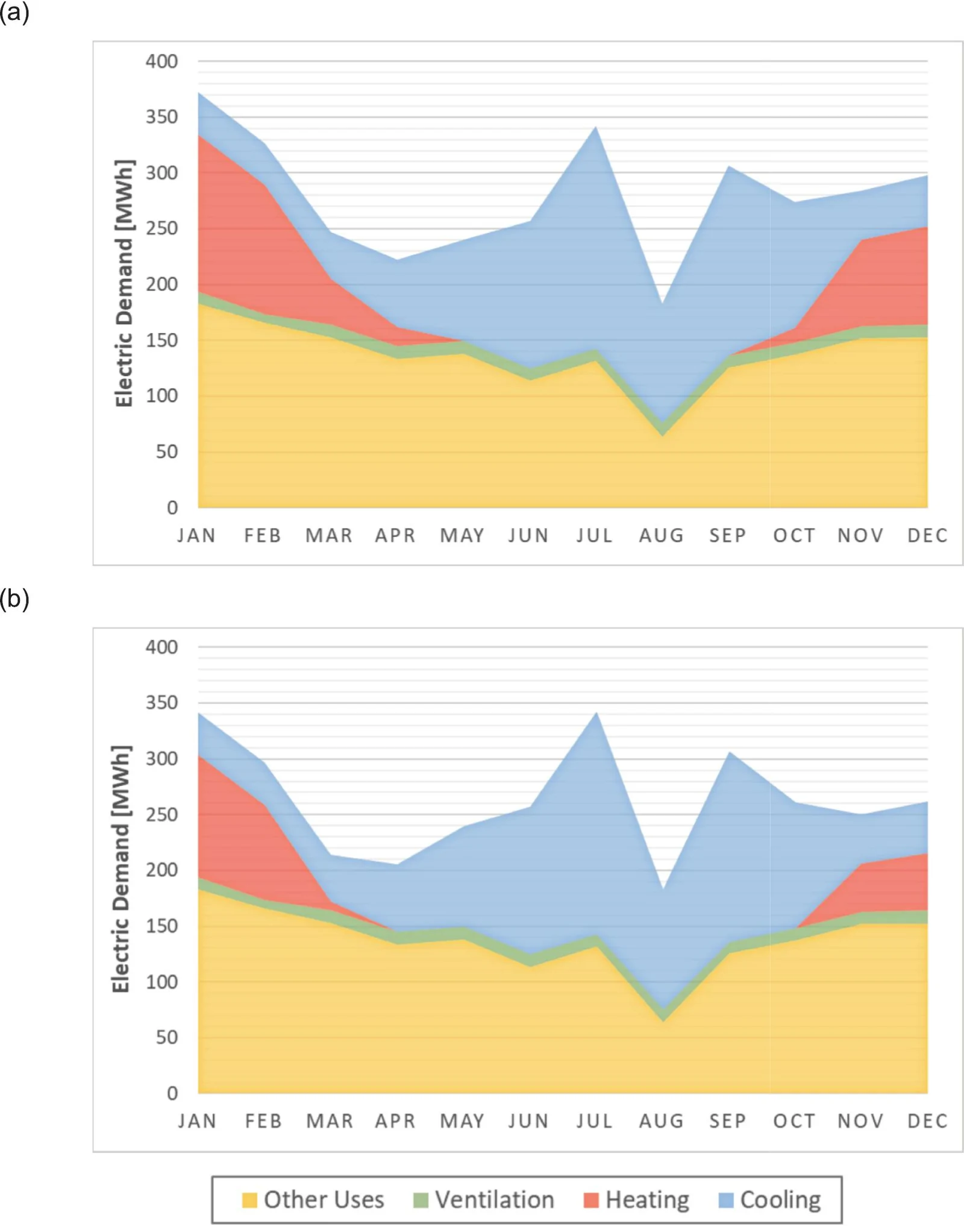
Monthly electrical demand under enhanced efficiency scenarios: (a) Electrification of fossil fuel-based consumption. (b) Previous case plus waste heat recovery from the data centre.

The transition from gas-fired boilers to HP results in an increased electricity demand to about 3.5 GWh annually, with the monthly profile illustrated in Fig. 4-(a). Concurrently, the recovery and utilization of waste heat from data center cooling substantially offsets this increment, restoring electricity consumption to levels comparable to the baseline scenario. However, this approach requires targeted analyses to optimize the exploitation of the surplus thermal energy depending on the end use.

The waste heat generated by the data center’s cooling processes is produced nearly continuously throughout the day. However, building heating demand exhibits distinct diurnal peaks, primarily in the early morning (typically 07:00–09:00), necessitating a thermal energy storage (TES) system to bridge this temporal mismatch.

The proposed TES solution comprises four insulated water tanks, each with a 5,000-liter capacity, collectively storing up to 464 kWh of thermal energy for on-demand morning delivery. Detailed dimensioning calculations for this system are provided in Amador [58].

In contrast, greenhouse heating requirements predominantly occur overnight, aligning more closely with the data center's cooling cycle and waste heat availability. This natural synchronization facilitates higher utilization efficiency, although comprehensive modeling is recommended to optimize full integration if this aspect were to be analyzed in detail.

Finally, these combined interventions result in a net annual electricity consumption of approximately 3.15 GWh, essentially unchanged from pre-intervention values, while eliminating natural gas use and its associated direct emissions. Post-implementation disaggregation reveals the following breakdown: ∼1 GWh for cooling, 0.3 GWh for heating, 0.15 GWh for ventilation, and ∼1.5 GWh for other end uses, as detailed in the monthly profiles of Fig. 4-(b).

#### 4.2.2 The stochastic perspective on system Definition

To perform a detailed energy balance assessment of the system's performance, two key aspects must be addressed. First, the consumption profiles of each distinct demand category require granular characterization, which in this study is conducted on an hourly basis to capture temporal dynamics accurately.

Second, given the emphasis on maximizing self-consumption, the existing solar generation capacity across the rooftops of the various buildings must be precisely quantified and integrated into the analysis.

**Fig. 5:**
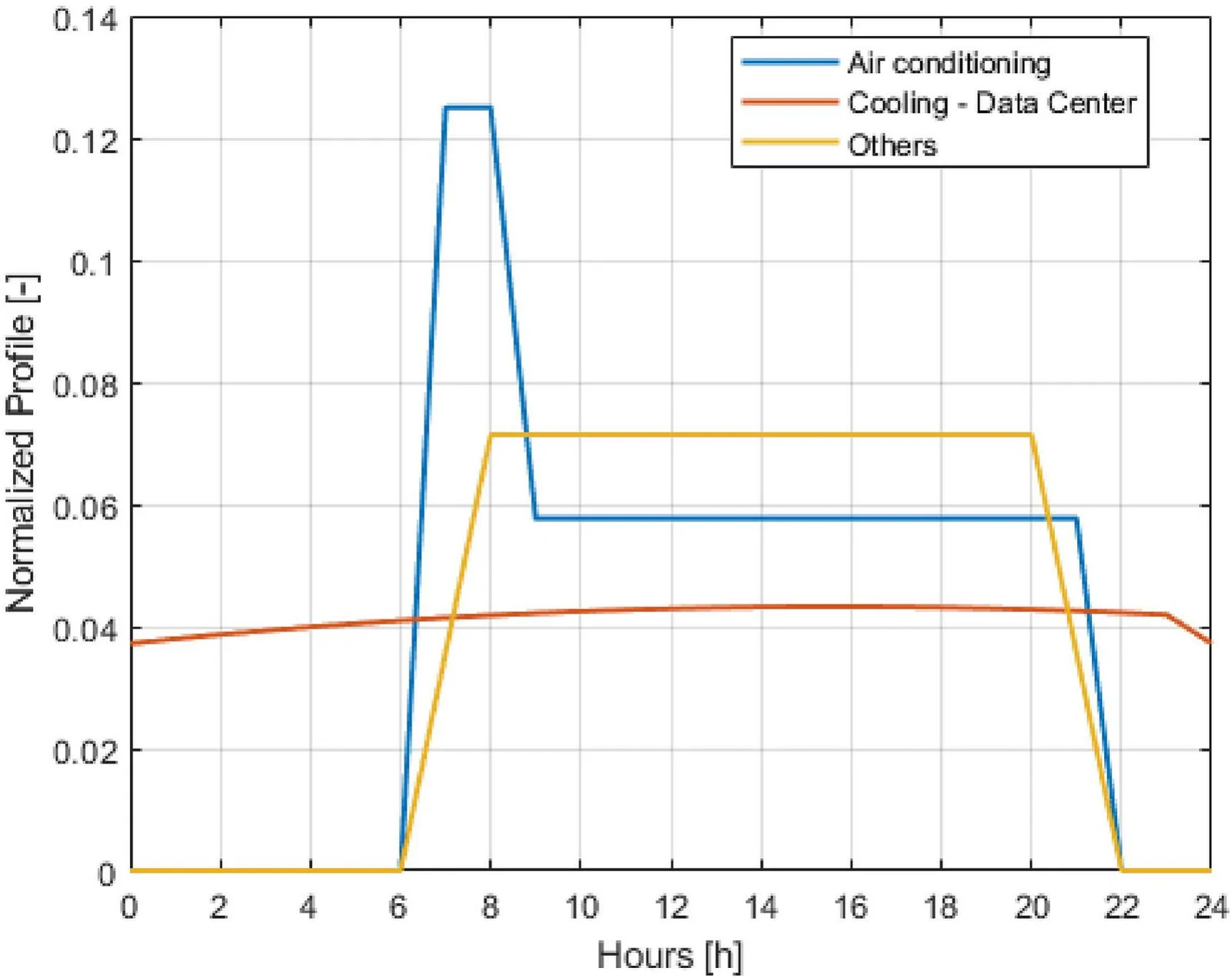
Normalized hourly profiles of the three principal consumption groups.

Fig. 5 illustrates the three main categories of electricity consumption considered in this study. Loads associated with air conditioning, namely heating, cooling, and ventilation, exhibit a characteristic diurnal pattern, with a pronounced peak at the beginning of the day corresponding to system start-up, followed by a stabilization as operating conditions are reached and maintained throughout the day. Notably, data center cooling (labelled as Cooling-Data Center) displays an almost constant profile over the entire year due to its continuous operation. The remaining consumption categories, including lighting, office equipment, and other end uses, show relatively stable daily profiles, although slightly reduced values are observed during early morning and late evening hours, reflecting lower occupancy and activity levels. For simplification, greenhouse cooling demand has been aggregated within the “Others” category. In contrast, the heating demand follows a different pattern. While it is initially supplied by gas boilers, in the optimized system configuration, it is entirely covered by excess heat recovered from the data center. As a result, heating demand does not appear as an independent load in the final modeling.

Finally, the potential for solar PV power generation on the rooftops of the analyzed buildings was estimated by comparing renewable output against prevailing thermal and electrical demands to quantify reductions enabled by the proposed efficiency measures. The objective is to maximize self-consumption by integrating solar energy and thermal storage, thereby enhancing building energy autonomy. Rooftop assessments for each ETSII building followed the methodology outlined by Hurtado-Pérez et al. for the UPV campus. [27], accounting for available surfaces and constraints such as obstacles (e.g., air-conditioning units, thermal ducts, lighting fixtures) and shading effects. Consequently, of the > 25,000 m^2^ total rooftop area, ∼12,000 m^2^ proved optimal for PV deployment, yielding a peak capacity of 753 kW [27].

Exploring recent agrivoltaics configurations, which entail installing PV systems over existing greenhouse roofs, unlocks an additional ∼6,000 m^2^ of viable area, thereby augmenting capacity by 541 kW, for a total of ∼1.3 MW of peak power. This approach aligns with emerging research on agrivoltaics and even full-site coverage with artificial lighting to sustain optimized crop yields. [61].

**Fig. 6:**
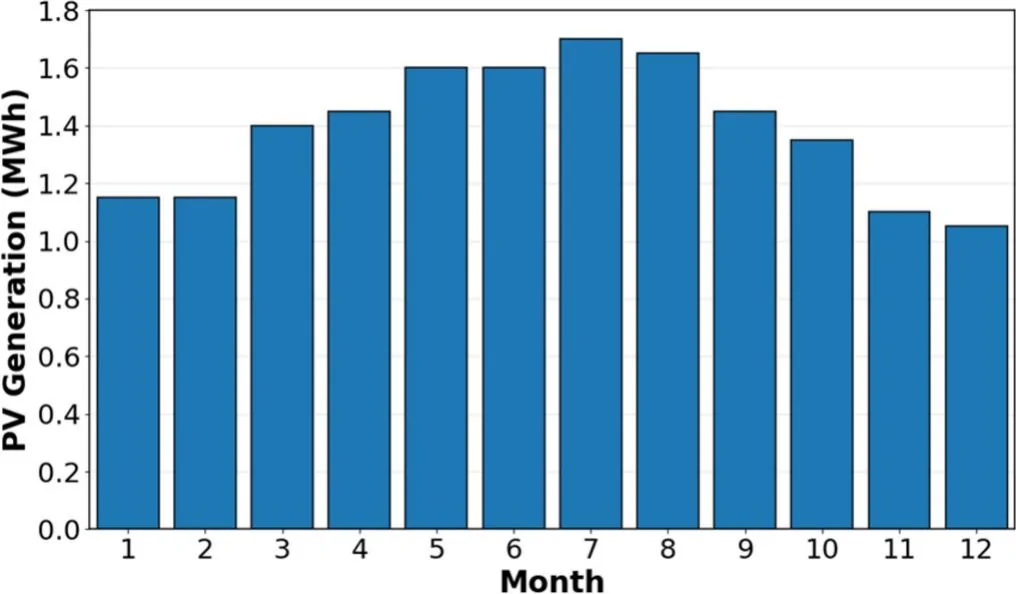
Monthly solar PV generation (1 MW PV installed, optimized slope and azimuth).

With the total installable peak PV capacity established, the next step entails estimating the hourly performance profiles of the proposed systems. Valencia's solar resource is characterized by an annual average irradiation of 1,735 kWh/m2/year, with pronounced seasonal variability: peaks in summer (June–July: ∼7.8 kWh/m2/day) and minima in winter (December–January: 2.1–2.5 kWh/m2/day), yielding a daily mean of 5 kWh/m2/day and a clearness index of 0.65. Accordingly, an optimized 1 MW PV array, employing site-specific tilt and azimuth angles, is projected to yield ∼1.6 GWh annually, as simulated using PVGIS and illustrated in Fig. 6 [56].

### 4.3 System assessment and uncertainty analysis

This section constitutes one of the central pillars of the study, providing a framework for evaluating performance under both deterministic and stochastic conditions. Phase 3 is divided into two subphases: deterministic and stochastic analyses. The deterministic subphase uses baseline data to construct a model that assesses system performance under defined operating scenarios, which include average variable values, typical profiles, and boundary conditions. The stochastic subphase extends this model by applying uncertainty quantification to input variables and delineating the range of system outcomes. Both analyses evaluate hourly energy balances between generation and demand. The deterministic analysis uses 2019 data as the reference year. The stochastic analysis incorporates over two decades of PVGIS historical hourly solar irradiance data to capture resource variability and recalculates corresponding demand profiles.

#### 4.3.1 Deterministic analysis of the System’s performance

The datasets outlined in the preceding section form the basis for a deterministic evaluation of the integrated energy system's performance. Hourly electricity generation from the proposed solar PV installations is modelled using “typical meteorological year” data, capturing site-specific irradiation patterns, to yield realistic production profiles. These are then reconciled against the hourly-resolved consumption distributions for each end-use category (e.g., cooling, heating, ventilation, and others), enabling a granular assessment of temporal alignment.

As a preliminary metric, an aggregate annual energy balance quantifies the total PV output relative to demand. A 753 kW peak capacity, deployed across ETSII rooftops, produces ∼1.055 GWh, satisfying roughly one-third (34%) of the baseline electrical demand (3.08 GWh). Augmenting this with agrivoltaics PV on ∼6,000 m^2^ of greenhouse roofs boosts total capacity to 1,295 kW, generating ∼2.1 GWh annually and covering nearly 70% of demand on an energy basis. Nonetheless, self-consumption efficiency is tempered by solar intermittency and mismatched diurnal/seasonal cycles; specifically, ∼68% (∼1.43 GWh) is directly utilized on-site, while the residual 32% (∼0.67 GWh) constitutes grid exports. These figures lead to a self-sufficiency rate of ∼46.5%. This comparatively high self-consumption fraction arises from robust synchrony between PV generation peaks (midday) and university activity patterns, which predominantly occur during daylight hours.

**Fig. 7:**
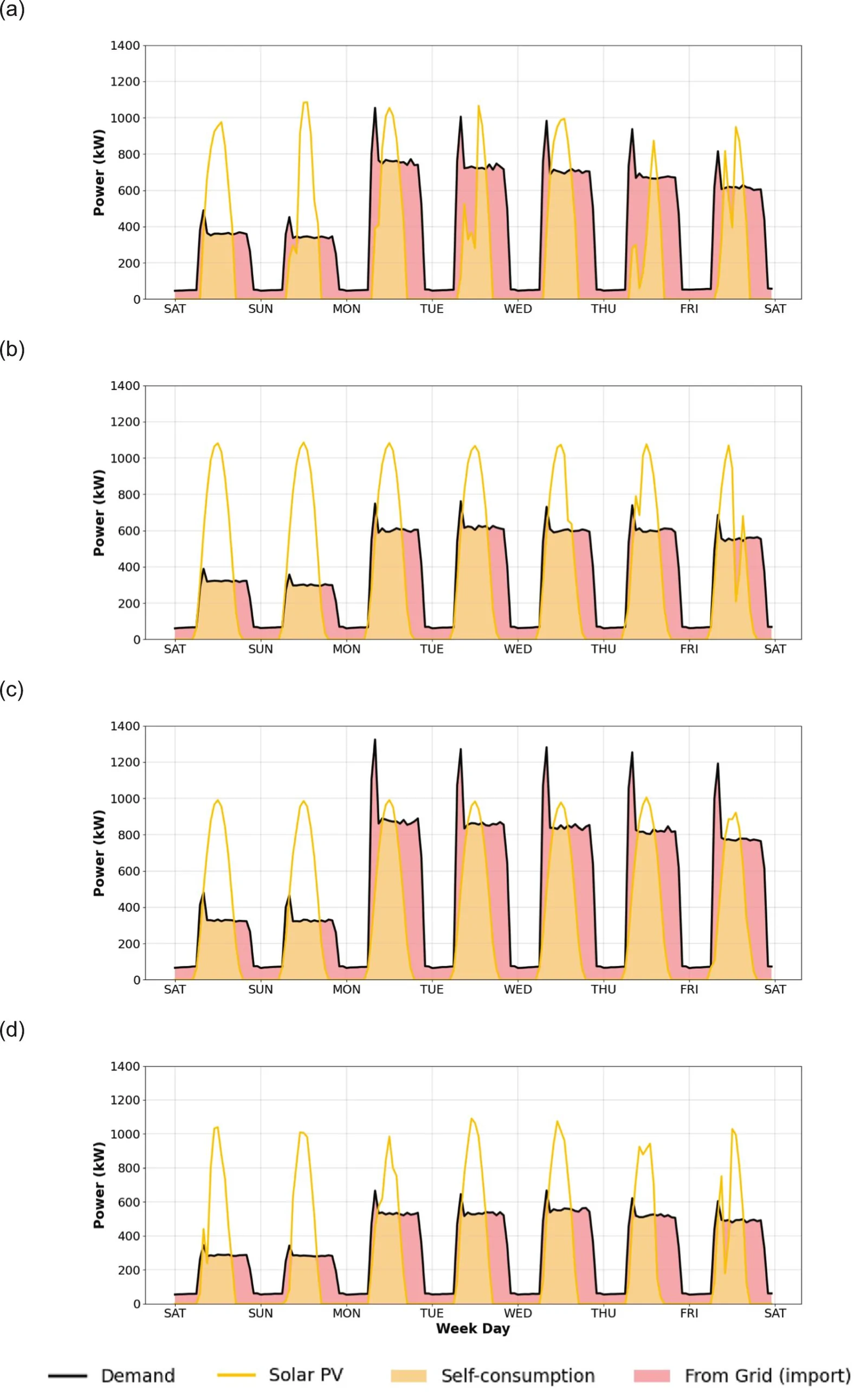
Energy balance at ETSII, UPV: (a) Winter; (b) Spring; (c) Summer; (d) Autumn.

For deeper insights into intra-annual dynamics, Fig. 7 presents normalized performance traces over four characteristic weeks, one for each season: Summer presents high insolation and high daytime, but solar panels present efficiency problems because of temperature); Spring has quite high irradiation and daytime, but this lower temperature problems; Autumn has lower insolation and daytime than Spring; finally Winter has the lowest insolation and daytime. Then, solar PV panels exhibit marked seasonal variations driven by irradiance and daylight duration in Valencia.

Spring (March-May) delivers higher Global Horizontal Irradiance (GHI) of 5.5–6.5 kWh/m^2^/d, longer daylight (12–14 h), and optimal solar elevation angles, yielding PV outputs 30–50% above annual averages with efficiencies near 18–20% due to moderate temperatures. Summer (June-August) has the best GDI and longest daylight, but temperature effects on solar panels reduce efficiency below that of spring. Autumn (September-November) sees reduced GHI (4–5.5 kWh/m^2^/day), shorter days (10–12 h), and lower angles with rising cloudiness, cutting performance by 20–35% despite similar mild temperatures that limit thermal derating. Winter (December-February) features minimal GHI (2–3.5 kWh/m^2^/day), the shortest days (9–10 h), and low angles with occasional clouds, which significantly reduce generation performance compared to spring. However, moderate temperatures (rarely below 10°C) preserve efficiency.

#### 4.3.2 Stochastic analysis of the System’s performance

Despite the deterministic analyses outlined previously, actual system performance deviates due to uncertainties in both PV generation and demand profiles, necessitating a stochastic framework to quantify behavioral variability. Classical parametric statistical methods presuppose known population distributions (e.g., normal, exponential, log-normal), offering precision yet faltering when distributions remain unspecified or poorly characterized. Non-parametric alternatives elude this limitation by avoiding distributional assumptions, proving advantageous in domains like manufacturing and energy modeling where empirical unpredictability prevails. Monte Carlo simulation, probably the most typical non-parametric technique, propagates input uncertainties through thousands of randomized iterations to empirically derive output distributions, accommodating multifaceted variabilities at the expense of substantial computational overhead. Wilks' method provides a computationally efficient surrogate [[38], [55]], originating in the 1940 s, to construct tolerance intervals encompassing a coverage fraction γ (e.g., 95%) with specified confidence β (e.g., 95%) via minimal simulations.

**Fig. 8:**
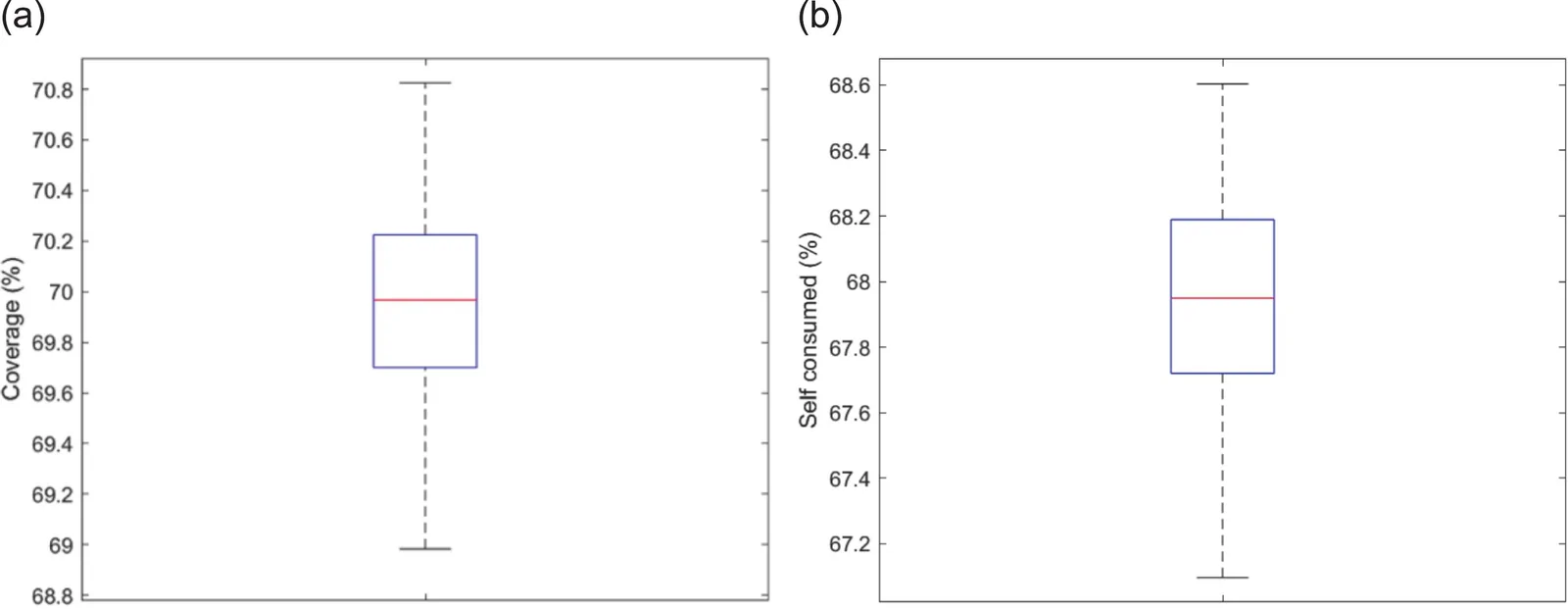
Percentage ranges at 95 × 95% coverage and confidence levels for the solar system: (a) Coverage; (b) Self-consumption.

Solar generation uncertainty has been modelled using 23 years (2001–2024) of PVGIS hourly data [56], with weekly beta-distributed synthetic profiles fitted to capture intra-day/seasonal irradiance fluctuations [31]. These facilitate probabilistic assessments of shortfalls, grid exports, and storage optimization. Demand-side variability is analogously addressed through disaggregated consumption profile analyses. Therefore, by moving from the deterministic simulations described above to a stochastic framework, which explicitly propagates uncertainties between input variables (PV generation, demand profiles), the probabilistic limits shown in Fig. 8 are obtained.

Fig. 8-(a) delineates demand coverage intervals spanning ∼68.85–70.8% (remember that these values are for a 95%/95% coverage/confidence levels via Wilks' method). Complementarily, Fig. 8(b) quantifies self-consumption rates at ∼67.1–68.65% of generated output, implying grid export fractions of 31.35–32.9% of the generated energy. That is, the installation generates approximately 70% of the demanded energy (Fig. 8-(a)), and of that produced energy, just under 70% is self-consumed (Fig. 8-(b)), so overall, around 50% of the consumed energy is self-produced, with the remaining 50% imported from the grid. To further analyze the temporal dynamics of grid export patterns, Fig. 10 presents hourly energy exports and imports to the grid, averaged across the 93 Wilks-sampled stochastic scenarios over the full 8,760-hour annual cycle.

**Fig. 9:**
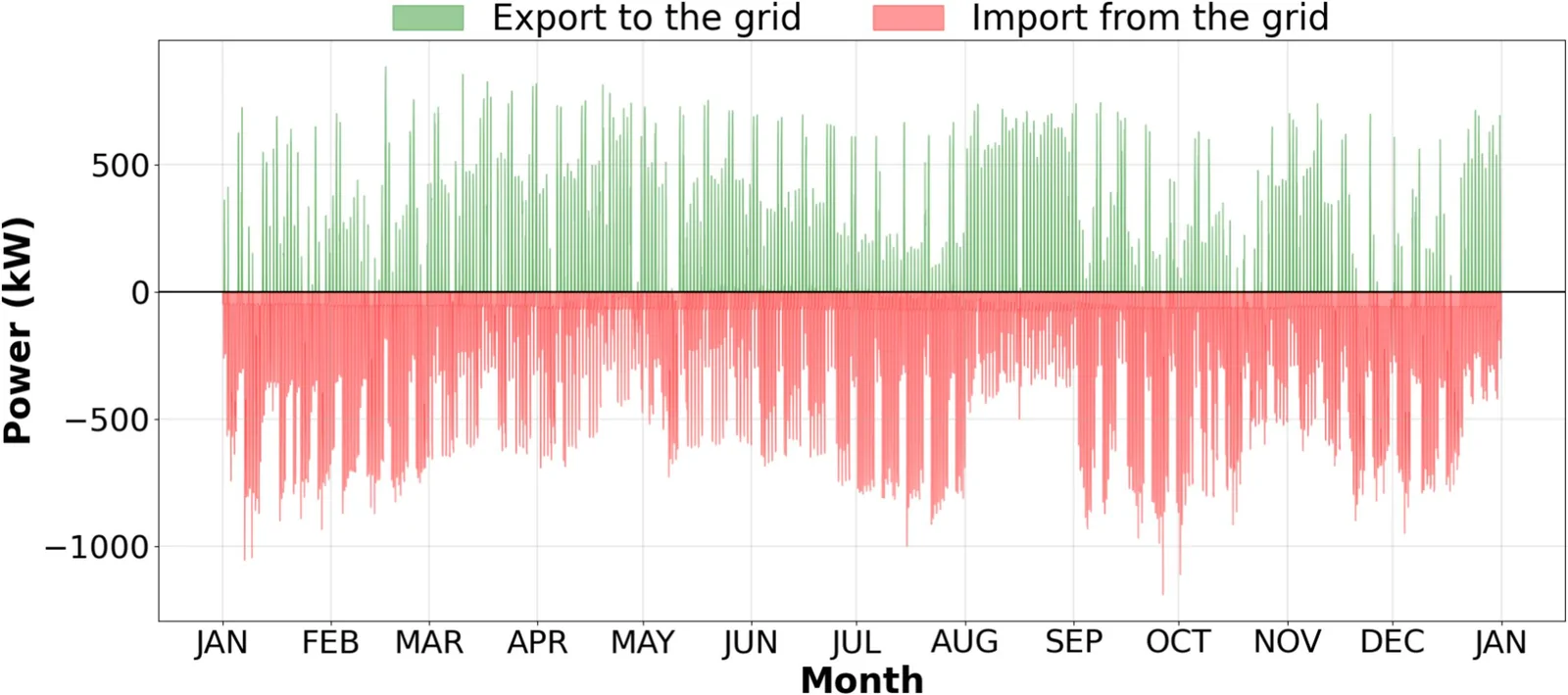
Energy exports and imports to the grid per hour over one year.

**Fig. 10:**
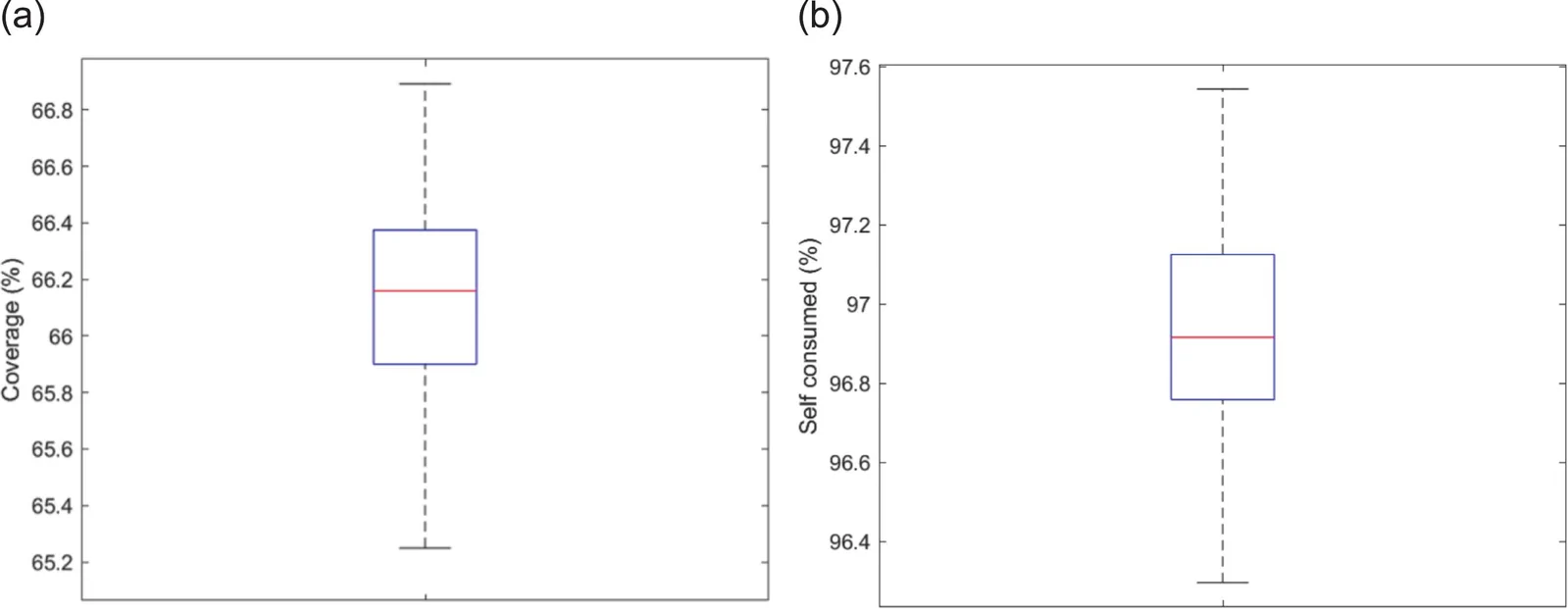
Percentage ranges at 95 × 95% coverage and confidence levels for the solar system with a mega-battery module: (a) Coverage; (b) Self-consumption.

For a more detailed analysis of the system's behavior throughout the year, Fig. 10 presents the average hourly exports and imports across the 93 simulated cases. Regarding exports (green lines of Fig. 9), prominent peaks repeat throughout, attributable to diminished weekend consumption and associated surpluses during midday PV generation. Notably, summer vacation periods (the whole month of August) exhibit markedly elevated exports due to sustained high isolation and minimal demand. Analogous surges occur during extended holidays, such as Fallas, Easter, and Christmas, highlighting seasonal operational mismatches.

Imports (red lines of Fig. 9) exhibit an approximately inverse pattern to exports. August shows markedly lower imports due to reduced overall consumption during summer vacations. Similarly, extended holiday periods, such as Fallas, Easter, and Christmas, feature diminished imports due to lower demand. Weekends consistently display reduced imports, reflecting lower activity levels. Spring exhibits particularly low imports, driven by enhanced PV generation efficiency and minimal heating or cooling needs in buildings (only data center cooling is required). Autumn presents a comparable but less pronounced trend, with moderate generation gains offset by slightly higher thermal demands than in spring.

Obviously, the substantial surpluses during extended vacation periods, particularly summer, cannot be feasibly utilized at competitive costs due to their duration and scale, rendering storage or alternative dispatch uneconomical. In contrast, weekend excesses are more amenable to mitigation, and even shorter vacation periods (e.g., Fallas, Easter) may prove viable for targeted interventions such as demand-side management or modest battery buffering. This distinction highlights opportunities for cost-effective strategies that are adapted to mismatched intermittency.

Consequently, as previously discussed, the presence of recurrent low-demand periods (weekends and vacation breaks) creates a clear opportunity to enhance the self-consumption ratio through the integration of an electrical energy storage subsystem. Specifically, since weekend demand is approximately half of that of working days, most surpluses are concentrated on weekends. Then, a storage system with an energy capacity on the order of one or two days of PV production and a power rating comparable to the peak generation capacity could, in principle, absorb these excesses and subsequently discharge them during working days. Thus, although the current configuration already exhibits a high degree of self-consumption due to the relatively strong alignment between generation and demand, the deployment of storage is expected to significantly increase this percentage.

To quantify this potential, the impact of battery storage on self-consumption was assessed using the largest commercially available Tesla model, the Megapack 2 XL [62]. This large-scale storage unit provides a charge/discharge power of 979 kW per module, an energy capacity of 3.916 MWh, and a round-trip efficiency of 93.7% in its 4-hour configuration. For the initial sizing analysis, a single module was considered, motivated by the observation that surpluses appear systematically on weekends and, to a lesser extent, during midday hours on working days. The ability to capture weekend surpluses is therefore expected to yield the greatest marginal gain in self-consumption.

Reapplying the uncertainty analysis with the inclusion of one battery module produces the demand coverage and self-consumption ranges shown in Fig. 10. As shown in Fig. 10-(a), overall demand coverage remains very similar to the no-storage case, with only a modest reduction attributable to storage losses (on the order of 3.5–4%). In contrast, the effect on self-consumption is substantial: the fraction of generated energy self-consumed increases from just below 70% (Fig. 8-(b)) to values exceeding 95% (Fig. 10-(b)), highlighting the strong synergy between storage and on-site solar PV generation.

In summary, the system produces about 66% of the total demand (Fig. 10-(a)), with around 97% of that production effectively self-consumed ((Fig. 10-(b), resulting in over 65% of the overall demand being met by the system itself. This reduces grid imports from roughly 50% in the no-battery case to about 35% when a battery module is added. Storage plays a key role in PV systems, as it enhances self-sufficiency and minimizes grid dependency, as demonstrated through deployment of one mega-battery module.

**Fig. 11:**
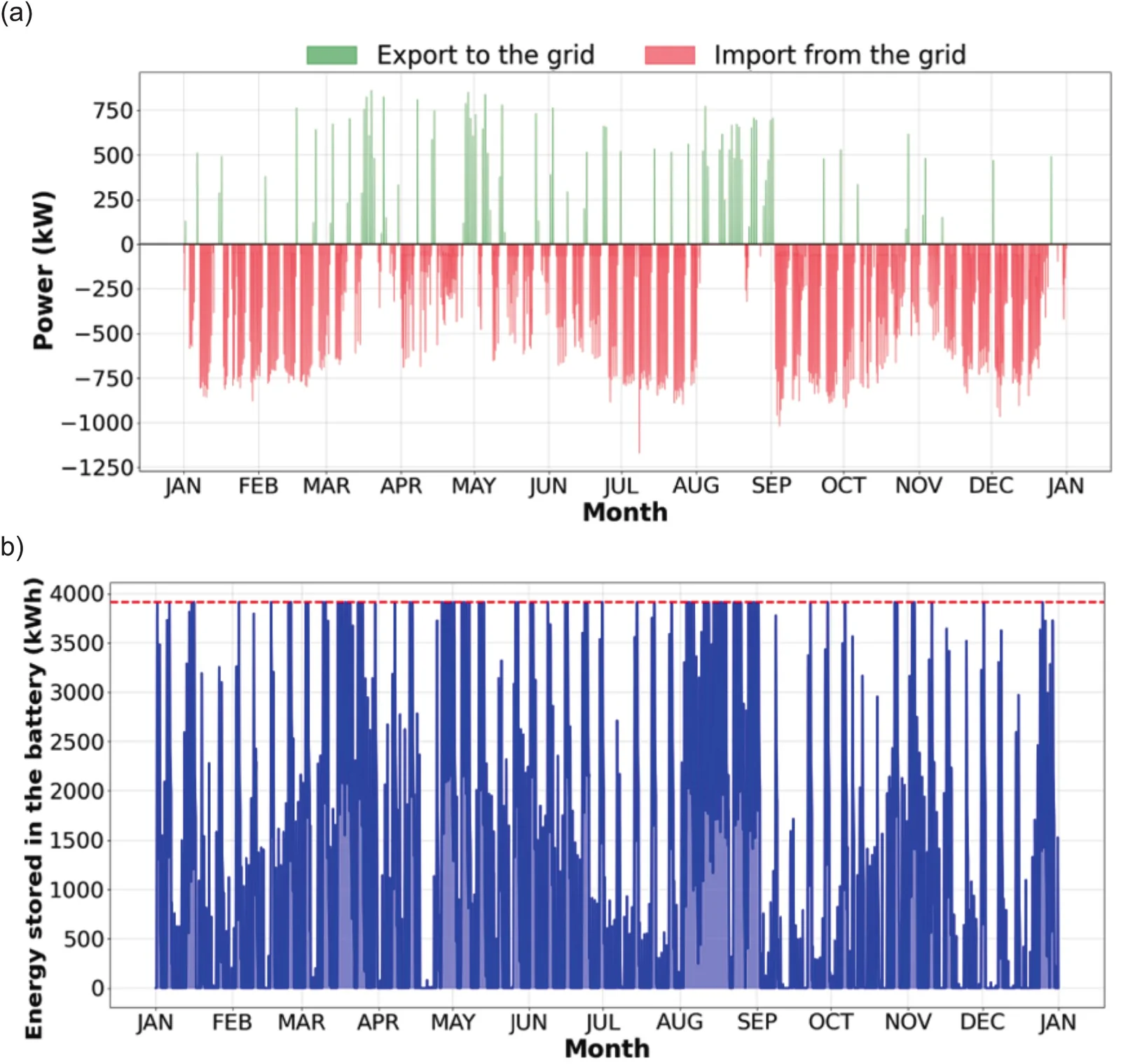
Hourly average performance of the random sampling for the solar energy system with batteries over one year: (a) Energy exports and imports to the grid (b) Energy stored in the battery.

Fig. 11-(a) illustrates the hourly profiles of grid exports (green lines) and imports (red lines), averaged across the 93 stochastic scenarios of BEPU analysis with a single battery storage module integrated into the system. Focusing initially on grid exports, these occur almost exclusively during the summer vacation period, supplemented by minor contributions during other mid- and long-duration holiday intervals. In contrast to the non-storage configuration (Fig. 9). This configuration exhibits exports throughout nearly the entire year, with peak curtailment predominantly in summer but also substantially in spring; consequently, the battery integration yields a marked improvement. This is evidenced by a substantial mitigation of grid exports across most of the year, concomitant with a self-consumption increase from under 70% to over 95%. These results highlight the battery system's effectiveness in improving temporal alignment between solar generation and demand, thereby minimizing curtailment and enhancing on-site utilization under uncertain conditions.

Battery integration substantially improves import coverage, raising system self-sufficiency from under 50% (no-battery baseline) to over 65% with a single megawatt-scale module. This benefit is evident when comparing Fig. 9 and Fig. 11-(a): during vacation periods, the battery system achieves near-self-sufficiency by effectively managing generation-demand imbalances, virtually eliminating grid imports. Over the remainder of the year, it consistently reduces import reliance across all periods, thereby enhancing overall resilience and minimizing external dependency under stochastic conditions.

Fig. 11-(b) illustrates the corresponding state-of-charge (SoC) evolution, demonstrating the battery's capacity to capture nearly all PV surpluses. Residual uncaptured excesses arise from two principal constraints: (i) instances exceeding the module's maximum charge power (979 kW) when PV output surpasses ∼1,300 kW peak; or (ii) full SoC saturation, predominant during summer vacations within depressed demand and protracted high insolation, as well as select spring/summer midday peaks. Thus, virtually all residual grid exports are confined to this extended low-demand period.

**Fig. 12:**
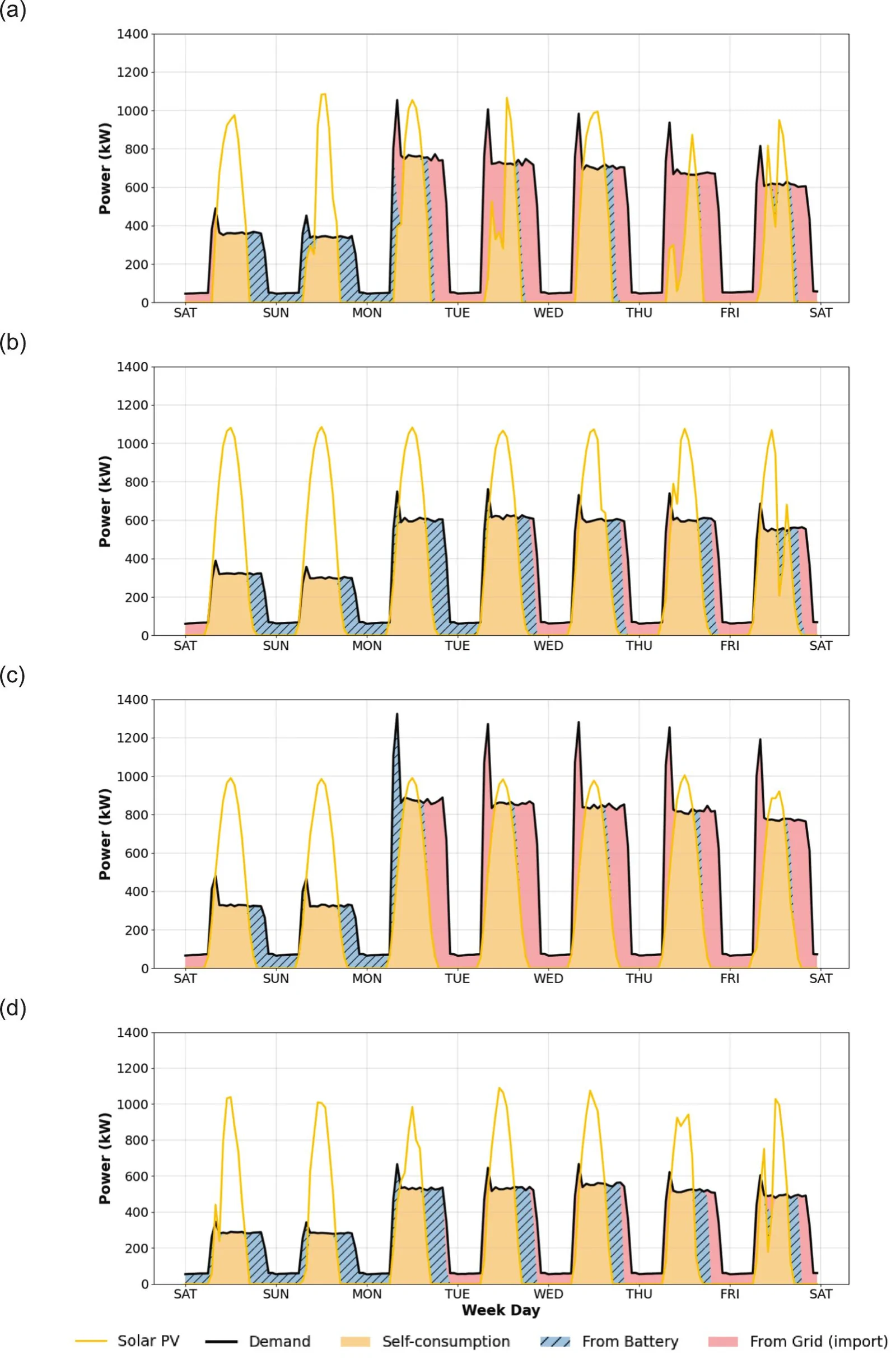
Energy balance at ETSII, UPV: (a) Winter; (b) Spring; (c) Summer; (d) Autumn.

**Fig. 13:**
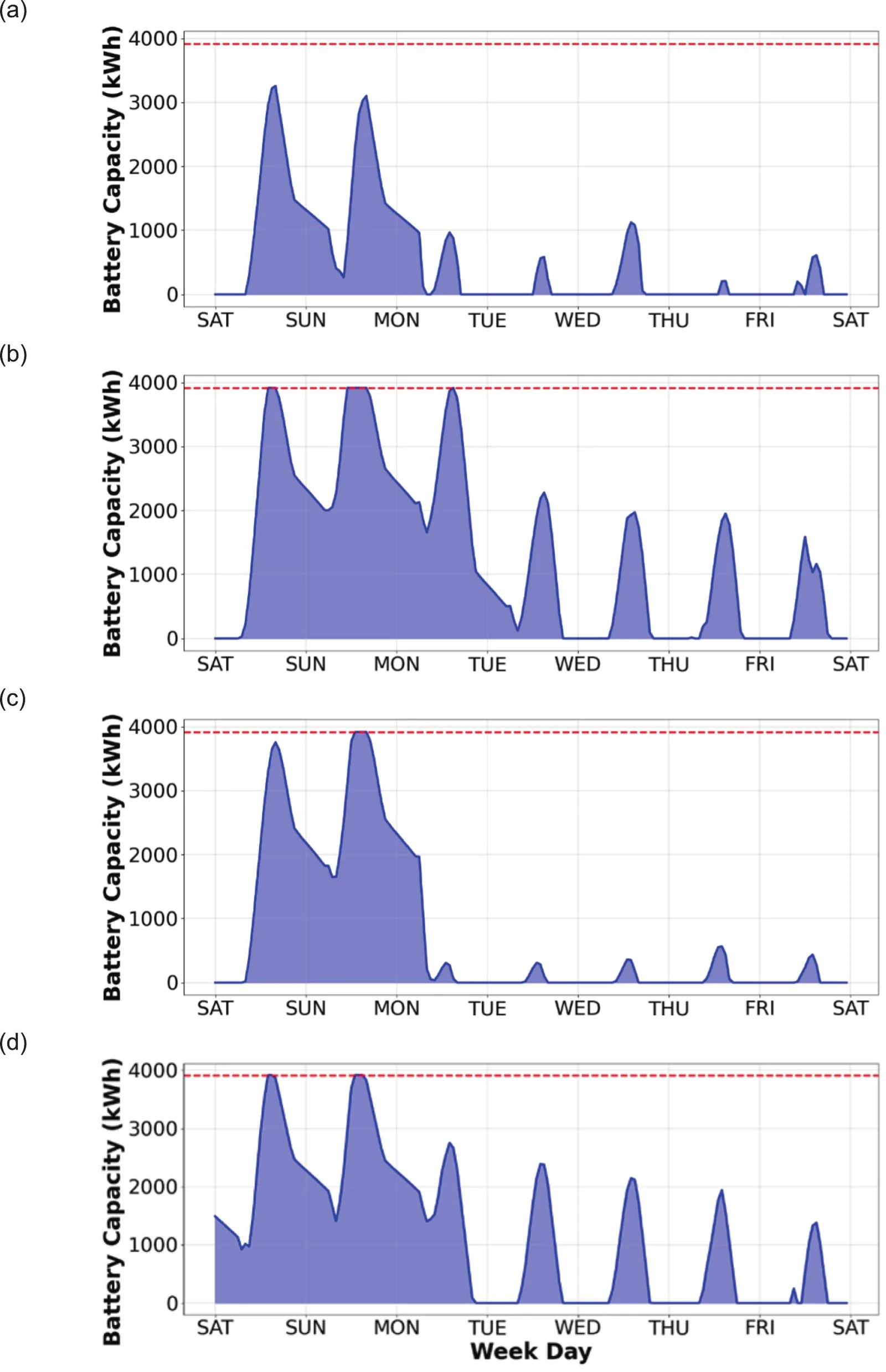
SoC for the proposed battery system of the ETSII at UPV: (a) Winter; (b) Spring; (c) Summer; (d) Autumn.

To offer a more detailed and insightful analysis of the system's performance with one battery module, Fig. 12 and Fig. 13 provide a more granular examination of system performance with battery storage across representative weeks from each of the four seasons. In the coldest months (Fig. 12-(a)), the system operates under its most demanding conditions, as this period corresponds to the lowest annual generation. Added to its lineup with high seasonal energy demand, mainly due to increased heating needs in end uses in buildings. During weekends (the first two days shown in the figure), the demand decreases significantly, allowing full demand coverage from Saturday morning onward, when generation exceeds consumption. As a result, a partial recharge of the battery module typically occurs, reaching a maximum SoC of approximately 75% by Sunday afternoon (Fig. 13-(a)). On the following weekdays, the midday solar PV peaks generally meet the demand; however, overnight the battery becomes fully discharged (Fig. 13-(a)) and cannot cover the nocturnal deficits, mainly due to the persistent loads of the data center. This results in significant coverage deficits, as shown by the pink trace in the figure. These gaps vary in severity; adverse weather, such as rain or heavy cloud cover, further reduces generation capacity, occasionally preventing the system from meeting even daytime or weekend demand.

More favorable conditions are observed in the mild and warm months, particularly during spring, when generation is considerably higher (Fig. 12-(b)). The demand is fully satisfied for most of the week, except for brief early morning deficits. During weekends, significant generation surpluses occur due to high PV output. In these cases, the batteries reach their full capacity (100% SoC), as shown by the first two charge cycles in Fig. 13-(b). This stored energy can sustain demand through the first working days of the week, up to late Tuesday, as illustrated in the example shown in the figure. Once the stored energy is depleted, partial midday recharges occur during the remaining weekdays, covering part of the nighttime needs. Nevertheless, reductions in solar irradiance caused by unfavorable weather conditions can disrupt this balance, leading to decreased storage, faster depletion of reserves, and consequently, poorer system performance.

The summer months exhibit a favourable operational pattern (Fig. 12-(c)). However, PV generation peaks remain lower than in spring due to elevated module losses from high ambient temperatures. These conditions coincide with the highest seasonal energy demands driven by intensified cooling requirements across building end-uses. The overall system behavior remains similar to that described for spring, as the extended daylight hours partially compensate for the lower generation efficiency. Accordingly, the system performance is slightly inferior to that of spring. The batteries reach full charge during the weekend (Fig. 13-(c)), and all demand is covered until early Monday afternoon, with similar trends observed on the remaining days.

Finally, autumn months (Fig. 12-(d)) represent an intermediate scenario between summer and winter. Reduced solar irradiance, shorter daylight duration, and relatively frequent adverse weather conditions collectively lead to a general decline in system performance. The storage unit often reaches full charge on weekends (Fig. 13-(d)), allowing stored energy to meet demand on the first working day. However, the typically unstable weather during these months appreciably deteriorates system performance.

**Fig. 14:**
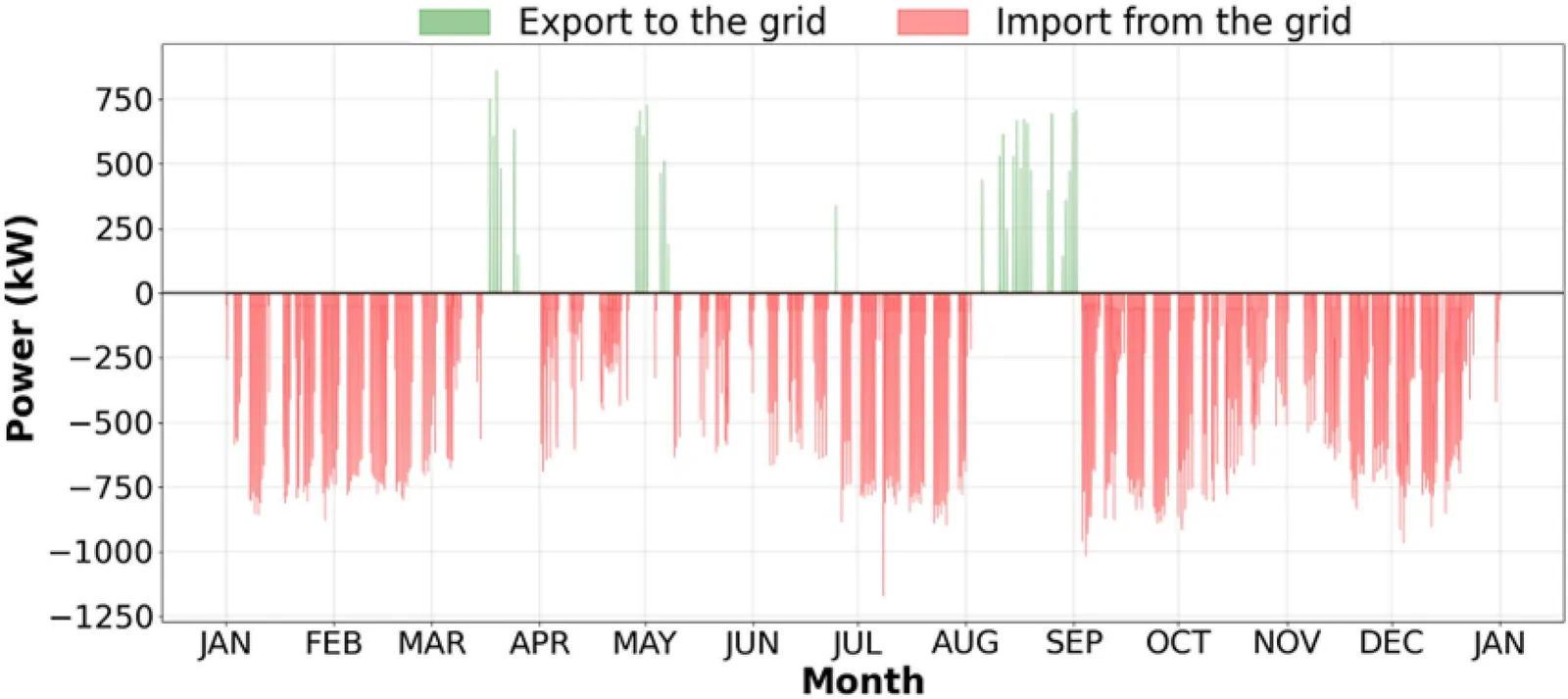
Energy exports and imports to the grid per hour over one year. Stochastic analysis with two battery modules.

In any case, a detailed evaluation of the system incorporating an additional battery module has been carried out, yielding very similar results, with only slight increases in self-consumption and subsequent slight reduction of grid imports (Fig. 14). Therefore, the results indicate that the addition of extra modules produces insignificant improvements in self-consumption rates. Then, the exploratory analyses confirmed no substantive improvements, consistent with observations in Fig. 13, which shows that the module attains full charge only transiently during warm-season weekends, limiting the utility of excess capacity for capturing residual surpluses, which occur infrequently.

In August, the main vacation month, self-consumption remains almost unchanged when adding extra modules, as adding them does not significantly change exports to the grid, since at the beginning of the period, the system would be fully charged, regardless of whether there are one or two modules. Marginal gains during brief holidays (e.g., long weekends, regional celebrations) prove insufficiently recurrent to warrant expansion, as do mid-duration breaks like Fallas (∼mid-March) and Easter (∼end-April).

As a summary, Table 2 displays the performance metrics of the proposed solar PV system under configurations without and with mega-battery storage modules, incorporating both deterministic and stochastic (Wilks-based) analyses.

**Table 2:** Summary of the key performance results for the solar PV system, without and with storage, with the deterministic and stochastic approaches.

Variables	Solar PV		Solar PV + 1 Batt.		Solar PV + 2 Batt.	
	Det.	Stoch.	Det.	Stoch.	Det.	Stoch.
Demand Coverage (%)	69.8	68.9–70.8	65.6	65.3–66.9	65.6	65.3–66.9
Self-Consumption (%)	68.3	67.1–68.7	96.7	96.3–97.6	98.7	98.3–99.7
Self Sufficiency Rate (%)	47.7	46.2–48.6	64.4	63.8–65.2	64.7	64.18–66.69
Energy fed to the grid (%)	31.7	31.3–32.9	2.79	2.45–3.75	1.30	0.27–1.67

The stochastic framework reveals performance variability attributable to fluctuating renewable resource conditions, though the annual solar irradiation and university demand profiles exhibit marked temporal stability. Consequently, tolerance intervals span less than 3% variability across key indicators: demand coverage, self-consumption rate, and grid exports.

Battery integration yields a significant benefit; a single module elevates self-consumption from ∼68–70% (without storage) to above 95%, approaching full utilization of solar PV production, albeit at the cost of losses of 3–4% of the total energy generated due to the round-trip efficiency of the battery system. It should be noted that the reference self-consumption rate of approximately 70%, which is exceptional for solar PV systems, is due to the strong synchrony between generation peaks and daytime demand, which is amplified during the warm months when university activities coincide with peak sunlight hours. The integration of a second module leads to slight improvements that are likely insufficient to justify its installation. Only a slight increase of approximately 2% in self-consumption is observed. However, under the most favorable conditions identified in the stochastic analysis, self-consumption can reach nearly 100%. Similarly, the excess energy exported to the grid is significantly reduced, becoming practically negligible. As shown in Fig. 14, surplus generation occurs only occasionally, primarily during long vacation periods, reaching values of around 1% of the total generation.

### 4.4 System’s strategic integration and results discussion

This subsection synthesizes the results obtained in Sections 4.1–4.3, extending beyond system characterization and performance evaluation to derive broader insights into energy system behavior, scalability, and policy relevance. The analysis integrates technical performance, uncertainty quantification, and implementation considerations to extract general conclusions applicable to similar urban and institutional contexts.

The results indicate that the ETSII campus exhibits a highly favorable alignment between electricity demand and solar PV generation, resulting in self-consumption levels of approximately 67–70% without the need for storage. This value significantly exceeds the typical ranges reported in the literature for building-scale PV systems, which generally lie between 30% and 50%, depending on demand profiles and system configuration [[63], [64]]. These findings reinforce and extend recent research highlighting temporal demand-generation synchrony as a key determinant of system performance. While previous studies have primarily focused on installed capacity and storage as the main drivers of PV efficiency, the present work quantifies the impact of intrinsic demand-generation alignment in an institutional context and demonstrates its implications for system sizing and design.

From the perspective of renewable integration, the system achieves approximately 70% annual PV coverage, again surpassing commonly reported values in urban and residential applications. However, consistent with well-established findings, high annual coverage does not translate directly into proportional self-sufficiency due to temporal mismatches between generation and demand. This confirms that time-resolved dynamics remain a critical limitation in renewable-based systems. The study contributes by explicitly quantifying this gap and showing, through stochastic analysis, that it remains remarkably stable under uncertainty, with variability below 3%.

The electrification strategy combining heat pumps and waste heat recovery confirms trends identified in prior research regarding emission reductions through sector coupling and heat recovery systems [60]. Several studies have shown that heat pump-based electrification, particularly when combined with heat recovery technologies, can substantially reduce emissions and improve overall system efficiency. However, unlike many analyses that report increased electricity demand following electrification, the current results show that total energy demand can remain stable when efficiency improvements and internal heat reuse are properly integrated. This provides additional evidence supporting the feasibility of demand-neutral decarbonization pathways.

The comparison between deterministic and stochastic results further reinforces these conclusions. Across all configurations, deterministic values consistently fall within narrow stochastic intervals, indicating that uncertainty introduces only limited dispersion and does not alter the overall performance trends. This suggests that, within the current framework, deterministic modeling offers a robust representation of system behavior, whereas the stochastic approach primarily serves to quantify uncertainty ranges rather than leading to fundamentally different results. In this context, uncertainty mainly affects operational margins, with only minor variations observed in indicators such as grid exports, particularly under storage configurations and during low-demand periods. This behavior contrasts with that commonly observed in other applications, both at the whole-grid level [5] and at the residential scale [65], where weaker generation-demand alignment generally leads to a greater sensitivity to uncertainty. In the present case, the high degree of temporal synchrony between generation and demand significantly reduces variability, enhancing system predictability and robustness.

The introduction of battery storage reveals a strong non-linear effect on system performance. A single storage module (approximately 1  MW of power and 4 MWh of storage capacity) increases self-consumption from around 70% to above 95%, while improving self-sufficiency from roughly 47% to 65%. These results highlight a key principle: moderately sized storage systems can substantially enhance renewable integration when effectively targeting recurrent short-term mismatches, such as weekend surpluses. However, the marginal benefit of additional storage capacity rapidly diminishes. Remaining excess generation is largely concentrated during prolonged low-demand periods (e.g., summer, Christmas, or Easter holidays), during which additional storage becomes economically inefficient given the limited potential for further improvement. This behavior highlights the importance of optimizing storage size instead of simply maximizing it, thereby avoiding oversizing driven by infrequent or extreme operating conditions. Moreover, these results outperform conventional building-scale studies, where storage systems typically achieve self-consumption levels of 80–90% (Liu et al. [66]). This improved performance is largely explained by the favorable alignment between generation and demand profiles at UPV, as well as in university environments more generally. Furthermore, the observed performance gains support the optimal sizing of battery systems at the 1  MW / 4 MWh scale and provide additional validation regarding the risks associated with oversizing identified in recent literature.

The stochastic BEPU-Wilks framework confirms that system performance remains highly predictable despite inherent uncertainties in solar generation and demand. This leads to another important conclusion: for systems with stable demand and well-characterized renewable resources, uncertainty primarily affects operational margins rather than overall system feasibility. Consequently, robust design can be achieved with relatively limited computational effort using non-parametric approaches.

Seasonal and temporal analyses further reveal distinct operational regimes. Winter performance is constrained by low solar availability and high demand, leading to deficits even with storage. Spring and early summer offer optimal conditions, with near-complete demand coverage and efficient storage utilization. In contrast, extended summer vacation periods produce persistent surpluses that cannot be economically absorbed. These results support a broader behavioral pattern: renewable-based systems are governed by a combination of short-term (daily/weekly) and long-term (seasonal/occupational) mismatches, requiring differentiated management strategies.

From a system design perspective, the results indicate that integrated solutions combining electrification (HP), renewable generation (PV), and selective storage offer a cost-effective and scalable pathway to decarbonization, particularly in institutional environments. The ETSII case demonstrates that high levels of decarbonization (full fossil fuel displacement and > 65% renewable self-sufficiency) can be achieved without excessive infrastructure expansion, in contrast with typical urban systems that require significant oversizing [63].

Finally, beyond the technical dimension, the study stresses the relevance of institutional-scale implementations as real-world testbeds. The UPV campus functions as a living laboratory, enabling the validation of scalable energy solutions and supporting their transferability to broader urban contexts. The proposed methodological framework, incorporating uncertainty analysis, provides robust and policy-relevant evidence to support decision-making in the context of energy transition strategies.

Building on the previous insights, the results allow the identification of several generalizable conclusions:

• Full electrification of energy uses enables deep decarbonization without increasing overall energy demand.
• Complementary strategies, such as waste heat recovery, further enhance system efficiency during this transition.
• Generation-demand synchronization is a key factor determining the performance of solar PV self-consumption, often outweighing the role of installed capacity.
• High degree of generation-demand temporal alignment significantly reduces the need for energy storage and the system’s performance sensitivity to uncertainty.
• Battery storage delivers maximum value when optimally sized to address recurrent short-term mismatches, with rapidly diminishing returns under extreme or infrequent conditions.
• Temporal variability, rather than annual energy balance, governs overall system effectiveness and must be explicitly considered in system design.
• In systems with stable demand and highly predictable or reliable energy resources, uncertainty primarily affects operational margins while having a limited impact on overall feasibility.
• Strong generation-demand alignment in solar PV systems leads to limited uncertainty propagation and high predictability, resulting in low variability in key performance indicators.
• This behavior is not directly transferable to other renewable technologies, such as wind generation, which typically exhibit higher intrinsic unpredictability and unreliability and therefore greater sensitivity to stochastic effects.

These findings position the ETSII campus as a replicable model for urban and institutional decarbonization, demonstrating how integrated, data-driven approaches can deliver robust and scalable energy transitions.

## 5 Conclusions

Decarbonizing urban-scale energy systems requires ambitious targets, with universities serving as leading institutions to develop demonstrator projects that validate scalable implementation pathways.

Two primary strategies facilitate GHG mitigation at building-cluster scales: enhanced energy efficiency and optimized renewable self-consumption. An integrated methodology combines efficiency audits with solar PV deployment, initially applied as a pilot to ETSII buildings at UPV for prospective campus-wide extension. Full electrification forms the foundation, substituting fossil fuel thermal loads with HP powered by on-site PVs, augmented by data center waste heat recovery to meet seasonal heating requirements.

System design addresses two key uncertainties: PV generation variability (resulting from irradiance and temperature effects) and demand fluctuations (driven by occupancy patterns). Stochastic analyses using Wilks' tolerance intervals (95%/95% coverage/confidence) convert deterministic benchmarks into probabilistic limits. For the ETSII buildings' ∼3.1 GWh annual demand and 1.3 MWp PV array, deterministic modeling indicates ∼69% demand coverage (∼68% self-consumption, 32% grid export); under uncertainty, these yield 68.9–70.8% coverage and 67.1–68.7% self-consumption intervals, confirming stability within ∼3% variability.

A single Tesla Megapack 2 XL module (979 kW power, 3.916 MWh capacity, 93.7% round-trip efficiency) increases self-consumption to 96.3–97.6%, despite ∼3.5–4% energy losses, by addressing weekend and vacation generation-demand mismatches. A second module yields only ∼2% additional self-consumption gain, insufficient to warrant deployment.

This framework highlights the propagation of uncertainty for robust energy system evaluation and validates efficient electrification, PV, and storage sequences for low-carbon transitions. In systems exhibiting high baseline self-consumption due to daytime demand-PV alignment, technical viability improves significantly. Beyond deterministic single-point estimates, the stochastic approach provides statistically bounded performance ranges that improve design robustness and support more reliable engineering decisions regarding PV sizing, storage capacity, and oversizing prevention. Pilot outcomes inform policies targeting energy autonomy through renewable-dominant configurations with moderate storage.

The ETSII-UPV study, guided by this methodology, serves as a reference case for advancing regulatory frameworks and enhancing stakeholder awareness in Spain's energy transition. Universities demonstrate institutional leadership in advanced PV self-consumption and integrated decarbonization. Ambitious decarbonization objectives, aligned with the European Green Deal and national 2050 pathway, require viable technical solutions alongside robust regulatory mechanisms for scaling deployment. These objectives also necessitate awareness among stakeholders, including regulators, administrations, enterprises, citizens, and academia, to promote social acceptance and shared responsibility. The methodology incorporates benchmarking of established practices, high-resolution hourly data acquisition, regulatory alignment, and early stakeholder involvement. This validates specific interventions' technical–economic feasibility while generating transferable principles for regional and national policies, consistent with European and domestic directives. From a practical perspective, the proposed framework can support energy managers in prioritizing electrification measures, sizing PV and storage systems under realistic variability.

However, the present study has some limitations, which open the way for several future research lines addressing issues such as degradation, tariff evolution, and demand-response operation. In this context, the applicability of the methodology could be extended and the evaluation of the system could be improved across diverse contexts:

- Techno-Economic Optimization. Quantify LCOE, payback periods, and NPV across environmental and financial metrics. The present analysis focused on emission reductions and autonomy, excluding capital/operational expenditure modeling.
- Lifecycle Degradation Assessment. Model long-term performance decay in PVs (∼0.5%/year), batteries (∼2–3%/year capacity fade), inverters, and balance-of-system components under local climate conditions for 20–25-year projections.
- Scalability Analysis. Apply the framework to diurnal-dominant facilities (such as offices and schools) that benefit from demand-PV synchrony and adapt it for nocturnal profiles (such as residential and industrial areas) via site-specific irradiance-demand characterization, accounting for modulated storage requirements.
- Advanced Storage and Demand Response Modeling. Incorporate optimized battery dispatch, flexible loads, EV charging management, and demand-response strategies to evaluate how operational control can further improve self-consumption, self-sufficiency, and grid interaction under uncertainty.

## CRediT authorship contribution statement

**César Berna-Escriche:** Writing – review & editing, Writing – original draft, Validation, Supervision, Software, Resources, Project administration, Methodology, Investigation, Formal analysis, Data curation, Conceptualization. **Lucas Álvarez-Piñeiro:** Writing – review & editing, Writing – original draft, Visualization, Validation, Supervision, Software, Resources, Methodology, Investigation, Formal analysis, Data curation, Conceptualization. **Paula Bastida-Molina:** Writing – review & editing, Visualization, Validation, Supervision, Resources, Project administration, Methodology, Investigation, Funding acquisition, Conceptualization. **David Blanco-Muelas:** Writing – review & editing, Validation, Supervision, Software, Resources, Methodology, Investigation, Formal analysis, Data curation, Conceptualization.

## Declaration of Competing Interest

The authors declare that they have no known competing financial interests or personal relationships that could have appeared to influence the work reported in this paper.
